# Analysis of care and gender stereotypes in nursing scientific research: a scoping review

**DOI:** 10.1590/1980-220X-REEUSP-2024-0066en

**Published:** 2024-09-09

**Authors:** Gabriela Duarte Almeida Mundim, Maria Raquel Gomes Maia Pires, Maria Verônica Sousa Torres, Aline Oliveira Silveira

**Affiliations:** 1 Universidade de Brasília, Centro de Estudos Avançados Multidisciplinares, Brasília, DF, Brazil.; 2 Universidade de Brasília, Faculdade de Ciências da Saúde, Departamento de Enfermagem, Brasília, DF, Brazil.

**Keywords:** Nursing Care, Gender Identity, Nursing, Feminism, Atención de Enfermería, Identidad de Género, Enfermería, Feminismo

## Abstract

**Objective:**

To map evidence about care and gender stereotypes in nursing scientific research.

**Method:**

A scoping review developed under the JBI framework with analysis of gender perspective in care approaches. The searches were carried out on January 31, 2023 in SciELO, Scopus, CINAHL, PubMed, BDENF.

**Results:**

Of the 3,743 studies located, 25 were included. Evidence was grouped into categories: essentially female care (n = 9; 36%); calling and service of love (n = 3; 12%); erasure of gender inequalities (n = 2; 8%); “inadequate and harmful” care (n = 5; 20%); neutralization of gender and bodies (n = 3; 12%); and reporting oppression in care work (n = 3; 12%).

**Conclusion:**

Most scientific research on care reproduces gender stereotypes that reinforce the oppression of women in nursing. In contrast, resistance denounces naturalization of care as “inadequate and harmful”, for perpetuating gender oppression in care work.

## RESUMEN

**Objetivo:**

Mapear evidencias sobre cuidados y estereotipos de género en la producción científica de enfermería.

**Método:**

Revisión de alcance desarrollada en el marco del JBI con análisis de la perspectiva de género en los enfoques de cuidado. Las búsquedas se realizaron el 31 de enero de 2023 en SciELO, Scopus, CINAHL, PubMed, BDENF.

**Resultados:**

De los 3.743 estudios localizados se incluyeron 25. La evidencia se agrupó en las categorías: cuidado esencialmente femenino (n = 9; 36%); llamado y servicio de amor (n = 3; 12%); eliminación de las desigualdades de género (n = 2; 8%); atención “inadecuada y nociva” (n = 5; 20%); neutralización de género y cuerpos (n = 3; 12%); y reportando opresión en el trabajo de cuidados (n = 3; 12%).

**Conclusión:**

La mayor parte de la producción científica sobre cuidados reproduce estereotipos de género que refuerzan la opresión de las mujeres en la enfermería. En cambio, la resistencia denuncia la naturalización de los cuidados como “inadecuada y dañina”, por perpetuar la opresión de género en el trabajo de cuidados.

## INTRODUCTION

The “natural caregiver” stereotype, marked by issues of gender, class, race and generation, crystallizes the image of women as supposedly “designed” to care in view of their feminine nature, resulting in gender inequalities, especially in nursing. The label of natural caregivers reinforces the idea that care work would be intended for women, fixedly conditioned by feminine nature linked to biological determinism, despite being social constructions. From this perspective, if we take care as a central element for the realization of democracy, we see the unfair repercussions of this stereotype, including: precarious work relationships; difficulty accessing political spaces; triple working day; low pay; incipient social recognition; and expropriation of women’s time and energy, issues that imply greater injustices^(1,2,3)^.

It is well debated in the literature that care work – for children, older adults, sick individuals and housepersons – mostly carried out by women, whether nurses or not, is socially devalued, poorly paid (or not paid) and precarious. The most accepted conception of care work, originating from the sociology of emotions, concerns the constant and intense attention that one person pays to another’s well-being. Nursing, as a profession involved in care work, also faces unfair working conditions, especially in the care area. Recent research on the professional profile in Brazil highlights susceptibilities in the job market, such as devaluation with low wages, precarious relationships, multi-jobs, allocation difficulties, insecurity and violence in the workplace, among others^(4,5)^.

In nursing, stereotypes have marked the profession’s imagination and reality since its inception, imprisoning it in fixed adjectives that deepen inequalities in working conditions. Stereotypes can be understood as a prejudiced and generalizing view of characteristics that groups and individuals possess or attributes that society expects them to have. Stereotyping consists of ignoring a person’s unique characteristics and treating them as a mold. Review studies identify gender stereotypes in society’s views on nursing, including, for female nurses, presumed technical incompetence, poor academic and professional level, incipient autonomy and hypersexualization, and for male nurses, the questioning of masculinity, faces of the same injustice^(1,6)^.

Meanwhile, in the present study, we problematize how prejudiced views about the profession are fed back by our scientific discourses. In this regard, the studies that demonstrate the “natural caregiver” reissue in nursing studies is emblematic. The reasons for professional choice, for instance, continue to be marked by sexism, conservatism and idealizations of unattainable perfectibility, centered on a sanctified altruism of “being a nurse”. In turn, men in the profession demarcate their choices based on rational objectives, such as the possibility of employment and leadership in the category, clearly demarcating gender inequalities within the profession. In another study, sexualization of nurses, male leadership, women’s emotional fragility and care as a feminine attribute were interpreted as part of “society’s view of the profession”, with a lack of reflection on ideologized scientific discourses in nursing. As a result, we found an abyss between what nursing says and the profession’s daily life in an endogenous contradiction^(7,8)^.

In a critical counterpoint, the COVID-19 pandemic has exacerbated the extent to which the epithets of “angel” or “hero” nurses do not correspond to the harsh reality and the high mortality rate in the professional segment. A survey that investigated perceptions of these narratives among nursing professionals scientifically concluded how far the idealizations are from interviewees’ daily work. In Brazil, sentimental tributes from the media during the pandemic did not translate into the defense of minimum wage for nursing, which to date has persisted in the fight. Therefore, it is time to reflect on how much speeches we reissue in scientific studies contribute to such political fragility^(9)^.

This scenario reiterates the importance of investigating naturalization of gender stereotypes, understanding them as discursive manifestations of intricate power relations capable of rigidly limiting social practice and scientific research of nursing based on a supposed social determinism. The asymmetrical repercussions of naturalization of care – as a structuring character of gender inequalities that challenge women with skills based on an alleged causal linearity – justify the need to expand critical research about confronting naturalization of care as a feminine condition.

To analyze gender stereotypes, we previously carried out a theoretical study on feminist epistemology and care^(10)^, delimiting the following dimensions to the criticism of “natural caregivers” in nursing science: Gender – category of contestation for any binary meaning of man/woman restricted to biological sex which, in contrast, considers the power relations produced in performative and discursive acts about sex, sexuality, desire and gender in conformation of discriminatory heterosexual normativity^(11)^; Care work – everything we do for the well-being of someone or something, whether in the reproductive or productive sphere of life^(2)^; “Natural caregiver” – exclusive, unequal and unfair allocation of women to care tasks, in view of a supposed and immutable “feminine” nature^(1)^. Using the term “natural caregiver” in this study summarizes the many gender stereotypes linked to it in nursing.

Based on these assumptions, this article’s guiding question is: how is the evidence presented on approaches to care in nursing scientific research with regard to gender stereotypes? The study is justified by the centrality of care for nursing practice and the few scoping reviews that analyze gender perspective in these studies. Based on this premise, the objective is to map evidence about care and gender stereotypes in nursing scientific research.

## METHOD

### Study Design

This is a scoping review analyzing approaches to care in nursing scientific research from a gender perspective. This type of review aims to identify key concepts and knowledge gaps that can be deepened in future studies, based on the synthesis of evidence present in the literature^(12)^.

We carried out a scoping review on conceptions of care in nursing scientific research following the method recommended by the JBI^(12,13)^, with the stages: 1 – issue identification using the PCC mnemonic: P (Population); C (Concept); C (Context); 2 – inclusion criteria; 3 – two-phase research strategies; 4 – data extraction with analysis of conception of care from a gender perspective; 5 – systematization and presentation of results. The Prisma Statement 2020 (Primas-ScR) checklist recommendations were also used^(14)^. The protocol used in the study was developed and registered in the Open Science Framework under the link: https://osf.io/xv3ph/.

### Research Question

To construct the research question, we used the PCC mnemonic: P – nursing; C – care approaches; C –– gender stereotype; with delimitation of the question: how is the evidence presented on approaches to care in nursing scientific research with regard to gender stereotypes?

### Inclusion and Exclusion Criteria

Articles from scientific journals, available in full, that address care as an object of reflection in nursing, published by nurses and/or in nursing journals, were included. Studies that did not consider care as an object of discussion were excluded.

### Research Strategy

As the number of studies in investigated databases was sufficient to analyze the “natural caregiver” stereotype, we chose not to include gray literature in the search scope. For methodological rigor, we carried out an exploratory phase with the inclusion of keywords in Portuguese, English and French to investigate the relevance of descriptors, virtual nursing libraries and databases. In this phase, the search was limited from 2020 to 2021. Then, in the improvement phase, we expanded the search process, modified the descriptors, included only terms in English and adjusted the databases to progressively expand the investigative process. In this second phase, we did not establish limits regarding the period of publication or language, as we intended to investigate the scope of studies on concepts of care in nursing^(12,13)^.

When identifying articles relevant to the topic, we searched the following databases and/or libraries: SciELO, Scopus, CINAHL, PubMed, BDENF (Via VHL). Moreover, we performed a manual and reverse search in bibliographic references of identified articles. As for descriptors, we considered those recommended by the Medical Subject Headings (MeSH) of the National Library of Medicine (NLM), United States, as well as the Health Sciences Descriptors (DeCS). To increase the return, Boolean operators were used in this way: i – exploratory phase: (Enfermagem OR Nursing OR *Soins Infirmiéres* OR Enferm*) AND (*Cuidado* OR Care OR *Soins*) AND (*Gênero* OR Gender OR *Genero*); ii – improvement phase: (care OR practice) AND (gender) AND (nursing) AND (research OR studie). Chart 1 describes the search string performed on January 31, 2023.

**Chart 1:** Database search string and review phase – Brasília, DF, 2023.

Database	Search string	
	Exploratory	Improvement
SciELO	(ab:((*Enfermagem* OR Nursing OR *Soins Infirmiéres* OR Enferm*) AND (*Cuidado* OR Care OR *Soins*) AND (*Gênero* OR Gender OR *Genero*)))	(ab:((care OR practice) AND (gender) AND (nursing) AND (research OR studies)))
BDENF (Via VHL) in exploratory PubMed in improvement	((*Enfermagem* OR Nursing OR *Soins Infirmiéres* OR Enferm*)) AND ((*Cuidado* OR Care OR *Soins*)) AND ((*Gênero* OR Gender OR *Genero*)) Search limit: title, abstract, subject.	((care[Title/Abstract] OR practice[Title/Abstract]) AND (gender[Title/Abstract]) AND (nursing[Title/Abstract]) AND (research[Title/Abstract] OR studie[Title/Abstract]))
Scopus	(TITLE-ABS-KEY (*enfermagem* OR *enfermagem* OR *soins* AND *infirmiéres* OR enferm*) AND TITLE-ABS-KEY (*cuidado* OR care OR *soins*) AND TITLE-ABS-KEY (*gênero* OR gender OR *genero*))	(TITLE-ABS-KEY (care OR practice) AND TITLE-ABS-KEY (gender) AND TITLE-ABS-KEY (nursing) AND TITLE-ABS-KEY (research OR studie))
CINAHL	AB (*Enfermagem* OR Nursing OR *Soins Infirmiéres* OR Enferm*) AND AB (*Cuidado* OR Care OR Soins) AND AB (*Gênero* OR Gender OR *Genero*)	AB (care OR practice) AND AB gender AND AB nursing AND AB (research OR studie)

Source: Own preparation.

### Extraction of Results

Articles were pre-selected based on titles and abstracts, and the studies were then read in full. As a recommendation of the technique, screening in two stages (reading titles and abstracts; reading in full), data extraction and analysis of results were carried out independently by two evaluators. Disagreements were decided by a third party^(12,13)^. We used Zotero^®^ for reference management and Rayyan^®^ for decision-making in the screening phase. In extracting the results, Microsoft Excel^®^ made it possible to organize the studies by year, title, authorship, place of publication, language, methodology, objective and conception of care.

To analyze care from a gender perspective, we adapted the data extraction instrument recommended by JBI^(13)^ with the inclusion of the following questions, elaborated based on the gender^(11)^, care work^(2)^ and “natural caregiver” stereotype dimensions^(1)^: 1 – Does the article address the issue of gender from a feminist perspective and challenge the binary relationship? 2 – Do care approaches discuss power and gender relations intrinsic to care work? 3 – Does scientific research reflect on the repercussions of gender inequalities in nursing work? 4 – Does the article reissue moral values, discourses or practices that women were “born to care about”? In criticizing the stereotype, we considered studies that affirmatively address at least one of questions 1 to 3. For reissue, we classified those with a lack of discussion of gender and care work dimensions, with reaffirmation of “natural caregiver” stereotypes. For those who described the issue of inequality, without analysis or positioning, we considered a reissue.

### Analysis and Presentation of Results

In systematizing the results, we performed content analysis of articles with extraction of the respective empirical categories. To this end, we initially performed text skimming in full, highlighting excerpts considered relevant to the investigation regarding the reissue or criticism of gender stereotypes in nursing scientific research. We then produced spreadsheets in Microsoft Excel^®^ with the classification of the 25 articles according to answers to the four guiding questions prepared (1 – Does the article address the issue of gender from a feminist perspective and challenge the binary relationship? 2 – Do care approaches discuss power and gender relations intrinsic to care work? 3 – Does scientific research reflect on the repercussions of gender inequalities in nursing work? 4 – Does the article reissue moral values, discourses or practices that women were “born to care for”?). Each article was analyzed according to stereotype reissue or criticism, with excerpts from representative articles extracted to justify each answer to the guiding questions. From this first typification, we extracted six empirical categories from the selected content, three representing reissues and three that inform the criticism of gender stereotypes. The empirical categories extracted from the articles were as follows: Reissue: a) essentially feminine care; b) calling and service of love; c) erasure of gender inequalities. Criticism: d) “inadequate and harmful” care; e) neutralization of gender and bodies; f) reporting oppression in care work. For the purposes of greater objective visualization of evidence mapping and discussion, we chose to classify the number and percentages of the number of articles grouped into each of these categories. When presenting the results, we used the PRISMA flowchart, a table with the characterization of the 25 studies and another with the exemplification of excerpts representing the reissue or criticism of gender stereotype, depending on the case. In Chart 2, referring to the description of the 25 studies, we established identification codes (ID) for each of included studies numbered from S1 to S25.

**Chart 2:** Characterization of articles included in the scoping review by summarized title, place of publication, objective, participants and methodology. Brasília, DF, 2024.

ID	Title	Place and year	Objective	Participants	Methodology
S1	Learning to care: gender issues for male nursing students^(15)^	Canada, 1996	Reveal similarities and differences in the experiences of male nursing students.	20 male nursing students	Qualitative research
S2	The concept of care in male nurse work^(16)^	England, 2001	Analyze participants’ experiences and compare them with the literature on the concept of care in nursing practice.	8 male nurses	Qualitative research
S3	*Politicidade do cuidado como referência emancipatória para a enfermagem* ^(17)^	Brazil, 2005	Theorize the politicity of care and point out disruptive dynamics for nursing based on the care triangle.	Not applicable	Theoretical reflection
S4	*A questão do gênero no ensinar em enfermagem* ^(18)^	Brazil, 2009	Analyze the issue of gender in teaching care in nurse training.	21 female nurses, 13 from UEFS and 8 adult health professors	Qualitative research
S5	Nursing care from the perspective of ethics of care and of gender^(19)^	Colombia, 2013	Explore the ethical dimensions of concept and practice of care from a gender perspective.	11 nursing professionals (6 women and 5 men) who work at the Base Hospital in Valdivia, Chile	Qualitative research
S6	*Sexualidade e a interseção com o cuidado na prática profissional de enfermeiras* ^(20)^	Brazil, 2013	Analyze the intersection between sexuality and nursing care as a social practice.	09 nurses from Barbacena, Minas Gerais	Qualitative research
S7	*A politicidade do cuidado na crítica aos estereótipos de gênero* ^(1)^	Brazil, 2016	Analyze gender inequalities among Brazilian women in Portugal and in nursing.	Not applicable	Theoretical reflection
S8	*Aportes del enfoque de género en la investigación de cuidadores primário* ^(21)^	Spain, 2017	Review studies that incorporate a gender focus in care.	Not applicable	Qualitative research
S9	*La categoría de género en la investigación y producción de conocimiento en enfermería en Iberoamérica: aportes para el debate* ^(22)^	Mexico, 2017	Make gender biases or blindness visible in knowledge production in Ibero-American nursing.	Not applicable	Narrative review
S10	*Ser mãe e enfermeira: questões sobre gênero e a sobreposição de papéis sociais* ^(23)^	Brazil, 2017	Describe the experiences of nursing mothers in reconciling their social roles.	10 nurse mothers	Qualitative research
S11	*Cuidado ético do outro: contribuições de Edith Stein e Max Scheler* ^(24)^	Brazil, 2018	Analyze Edith Stein’s empathy and Max Scheler’s sympathy for ethical care for others.	Not applicable	Theoretical reflection
S12	*Emergencia del modelo de enfermería transmitido en las universidades españolas: una aproximación analítica a través de la Teoría Fundamentada* ^(25)^	Brazil, 2018	Know the meaning of the term nursing for teaching nurses at Spanish universities.	08 nurses teaching from Spanish universities (6 women and 2 men)	Qualitative research
S13	*Entre o Estado, a sociedade e a família: o care das mulheres cuidadoras* ^(26)^	Brazil, 2018	Investigate the care provided by family caregivers of dependent older adults and its social repercussions.	45 elderly caregivers (36 women and 9 men) supported in Home Care Services	Qualitative research
S14	*Estructura y organización de las representaciones sociales del concepto cuidar* ^(27)^	Mexico, 2018	Analyze the structure and organization of social representations of the concept of caring in caregivers.	38 caregivers of people with chronic illnesses (21 women and 17 men)	Qualitative research
S15	Gender and informal care: different sense and meanings for men and women^(28)^	Brazil, 2018	Examine the daily health care tasks of low-income women in northwest Córdoba.	56 low-income women	Qualitative research
S16	*La cuidadora familiar: sentimiento de obligación naturalizado de la mujer* ^(29)^	Spain, 2018	Make visible the role of family care restricted to women as part of gender roles motivated by a naturalized feeling of obligation.	09 female caregivers	Qualitative research
S17	Meaning of care before starting nursing professional training^(30)^	Cuba, 2018	Interpret the meaning of care for nursing students.	06 students enrolled in the first semester of nursing (4 women and 2 men)	Qualitative research
S18	The effect of gender role orientation on student nurses’ caring behaviour and critical thinking^(31)^	England, 2018	Explore the impact of gender roles on critical thinking and caring practices of nursing students.	449 nursing students who had at least one month of experience in clinical practice (310 women and 139 men)	Quantitative research
S19	Perception of caring among nursing students: Results from a cross-sectional survey^(32)^	Scotland, 2019	Analyze the perception of care among Spanish nursing students.	321 Spanish nursing students (200 women, 88 men, 33 no responses)	Quantitative research
S20	*Resistência e resignação: narrativas de gênero na escolha da enfermagem* ^(33)^	Brazil, 2020	Analyze narratives about the process of choosing higher studies of female students enrolled in nursing and pedagogy courses.	21 female university students from nursing, pedagogy and administration courses at private HEIs in São Paulo	Qualitative research
S21	The effect of gender-friendliness barriers on perceived image in nursing and caring behaviour among male nursing students^(34)^	England, 2019	Examine the relationships between nursing image, caring behaviors, and gender barriers experienced by male nursing students.	141 male nursing students who obtained at least 1 month of clinical practice experience	Quantitative research
S22	Burden and Gender inequalities around Informal Care^(35)^	Colombia, 2020	Understand the consequences of informal care for caregivers in a debate from a gender perspective.	Not applicable	Narrative review
S23	Paying the Caring Tax: The Detrimental Influences of Gender^(7)^	USA, 2020	Analyze gender inequalities arising from moral impositions on nurses’ care in the workplace.	Not applicable	Theoretical essay
S24	*Sentidos do cuidado para acadêmicos de enfermagem* ^(36)^	Brazil, 2020	Identify meanings of care for nursing students.	13 nursing students from the last period of graduation at a College of Nursing (no gender specification)	Qualitative research
S25	*Que não seja aquela enfermagem que pede silêncio* ^(37)^	Brazil, 2022	Analyze nurses’ sociopolitical knowledge in social movements.	6 female nurses involved in social movements and with political representation	Qualitative research

Source: Own preparation.

### Data Availability

As recommended by Open Science, the research data was deposited in a publicly accessible repository, under the link: https://doi.org/10.48331/scielodata.VKXGGD.

## RESULTS

**Figure 1:**
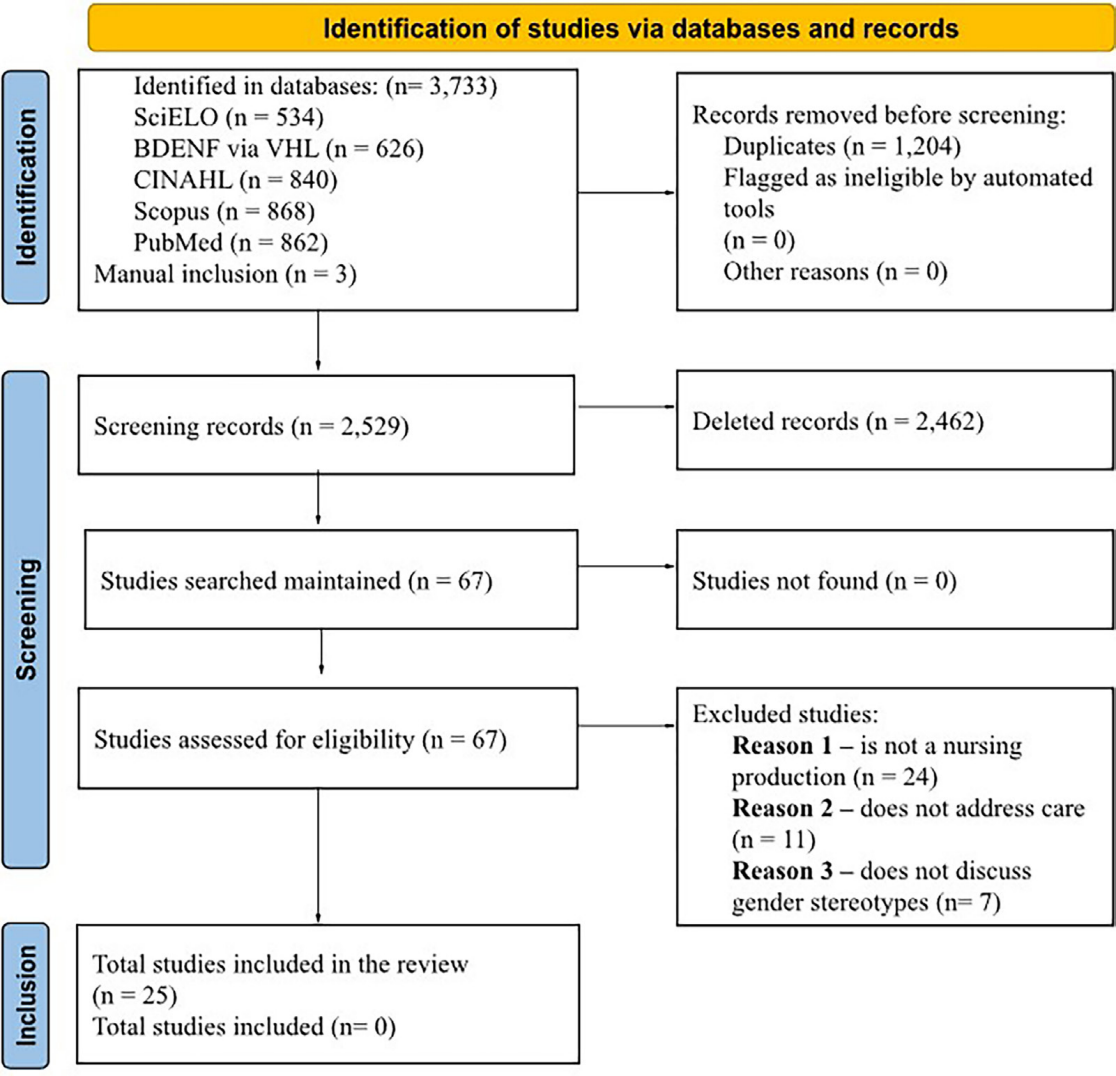
PRISMA study selection flowchart.

Source: PRISMA study selection flowchart.^14^

The search returned 3,743 studies which, with removal of duplicates, resulted in 2,529. In the first screening stage, by reading title and abstract, 2,462 articles were excluded. In the second screening stage, 67 articles were read in full and 42 were excluded for the following reasons: population was not nursing (n = 24); care was not the concept addressed (n = 7); and did not contextualize the gender stereotype (n = 11). The final sample consisted of 25 studies. Figure 1 presents the PRISMA^(14)^ flowchart of this review.

Chart 2 presents a description of the 25 articles included in the present review in relation to study identification, title, place and year of publication, objective, participants and methodology. Chart 3 analyzes the reissue or criticism present in studies regarding the “natural caregiver” stereotype, with examples of direct quotes taken from the respective articles. All translations carried out in these charts are our responsibility.

**Chart 3:** Analysis of care approaches in nursing scientific research included in the scoping review in relation to the “natural caregiver” stereotype according to results and exemplifying excerpt. Brazil, Brasília, DF, 2024.

“Natural caregiver” stereotype reissue	“Natural caregiver” stereotype criticism
S1: The study reinforces conceptions of binary gender restricted to two forms of care: a ‘masculine’ one, allegedly learned by male students; another ‘feminine’, supposedly natural for women. S1: “Freshman students […] admit that they learned aspects of care that ‘came naturally’ to their female colleagues […] An honest assessment of how women in nursing defined care as women’s work is needed […]”.^(15)^	S3: The thesis of the centrality of the political dimension of care is defended. It is argued in favor of a new logic of care, where assistance is exercised that, being power, both subjugates and emancipates. S3: “The politicity of care resides in the intrinsic ambivalence of help which, being power, both dominates and liberates human actions. […] It is about politicizing the social practice of nursing in the rich spaces where it operates, sharing decisions and expanding the debate around differences.”^(17)^
S2: The proposed conceptual model of care ratifies sexist gender stereotypes, namely, a more emotional profile for female nurses, in relation to the physical strength and virility of male nurses. S2: “Authors point out that a gender link is often made between care and femininity and that, increasingly, this is seen as necessary to transmit care to students”^(16)^	S4: Gender stereotypes related to care are evidenced and criticized in the speeches of teaching nurses, with directions for problematizing these issues in nursing training and practice. S4: “Understanding the relationships established between men and women who provide care will contribute to possible ruptures within nursing, which is undergoing a process of naturalization of its work, seen as an extension of domestic activities carried out by women.”^(18)^
S5: The study reproduces ideologies, moral values and gender segregation in the discourses of men and women in nursing regarding the ethics of care, with total erasure of inequalities. S5: “The ethics of care has positive characteristics that only human beings with a spirit of service can guarantee. Furthermore, there is no distinction of gender, ideology or race, which makes care a call to serve”.^(19)^	S6: Discusses the transversality of sexuality in nurses’ ways of caring. It reflects on nurses’ difficulties in dealing with situations that do not conform to heteronormativity. S6: “In the public space, the profession was, since the beginning of its institutionalization […], subjected to the strong scheme of neutralization of bodies and prohibition of sexuality. Christian morality, which even opened space for the belief in the nurse as an asexual angel, contributed enormously to the denial of nurses’ erotic body.”^(20)^
S10: The research describes the reconciliation between professional and family life of women nurses who are mothers, without, however, criticizing gender injustices arising from the overload of care work. S10: “In this professional practice, many components of the way of relating and the way of being feminine are mixed, which means that sensitivity and personal involvement with the suffering of others end up emerging within the scope of their professional activity […]”.^(23)^	S7: The politicity of care supported comparative analyzes of stereotypes of Brazilian Eves and Portuguese Marias with the sexualized or sanctified nurse. The gender inequalities of Brazilian women in Portugal and nurses are part of the Jewish-Christian morality that reiterates the subservience of the feminine to the masculine. S7: “One of these stereotypes that crystallize the imagination of women […] is the one that insists on designating them as a natural caregiver who, due to their feminine nature, would be destined to take full responsibility for care activities.”^(1)^
S11: Conception of care as a gesture of “love” full of moral and religious values present in the historicity of nursing, without questioning. S11: “Both Stein’s empathy and Scheler’s sympathy are closely linked to our human action as an act of perceiving the experiences of others, and [...] lead the sympathetic or empathizing human being to care for others with love.”^(24)^	S8: The systematic review identified weaknesses in the problematization of gender issues in 20 (57%) of the 35 (100%) articles that set out to analyze the relationship between care, gender and health. S8: “This situation requires problematizing the gender social order within a patriarchal context in which care is seen as a function associated with the domestic and highly feminized space”.^(21)^
S12: The authors identify and confirm in the speeches of nursing professors’ conservative ideologies, moral values and gender stereotypes that reinforce social injustices in the profession. S12: “Nursing can also be considered a quality that some people possess innately that leads to a special predisposition, called vocation, to help others [...] it is defined as a human activity linked to women and related to motherhood, the care of children and human survival. This maternal instinct is what provides the motivation and drive necessary to care”.^(25)^	S9: The review analyzes 104 nursing articles based on feminist epistemology. Gender prejudices and blindness are evident in scientific nursing research from countries in South America, Central America and Europe. S9: “This is one of the greatest problems in research […]: considering terms or concepts as invariable and fixed constructs. Especially in the profession, we must review the relationship between care and gender, in order to expand the fields of reading and open paths for alternative understandings of what apparently has not changed for so many years”.^(22)^
S14: Systematizes representations of informal caregivers who associate care with love, family, affection, protection or an acquired mission, reproducing the ideologies and gender stereotypes present in nursing. S14: “Caring for a sick person is a situation that women and men have experienced at some point in their lives, and that they naturally and spontaneously take responsibility, devote time and effort to others who cannot care for themselves due to age, illness, disability, or disability to meet their needs”.^(27)^	S13: The research reflects on the relevance of women caregivers for society and their invisibility for public policies, based on the care category, originating from feminist epistemology. S13: “[…] It is necessary to change the representation according to which the skills mobilized in care work are equal or a mere extension of the domestic functions socially attributed to women”.^(26)^
S15: The experiences of female caregivers are naturalized and essentialized, without critical reflection on gender issues. S15: “Women have learned the role of caregivers through years of socialization and have honed their skills by participating in the daily care of their children.”^(28)^	S21: It reflects on the implications of gender issues in the choice of nursing and pedagogy courses, resulting in professional devaluation. Ambiguities between resignation and resistance in students’ speeches point to perspectives for confronting injustices. S21: “In nursing […] feminization persists ‘both in university qualifications and at secondary and technical levels’ […] Thus, in the case of feminized health professions, the relationship between ‘care’ and ‘feminine action’ remains, in a process that naturalizes these differences as attributed to females”.^(33)^
S16: The study highlights the unequal division and precariousness of care work in the families surveyed, highlighting gender issues. However, it does not sufficiently problematize inequities, preferring to label as “superwomen” those who fully take on the multiple tasks. S16: “The term ‘superwoman’ […] perfectly defines these caregivers, who work hard to maintain multiple roles, including those of personal development, often interrupted due to an excessive workload […]”.^(29)^	S22: The research reflects on the overload of care tasks for women, resulting in gender injustices. Highlights the need to democratize responsibility for care within the scope of public health policies and the role of nursing in change processes. S22: “The fact that care has been, and continues to be, considered a topic within the feminine sphere, reinforces gender stereotypes about the role of men and women in society [...] Breaking with the roles socially designated for women in care could be the change that allows for a different distribution of care work.”^(35)^
S17: The research systematizes biological determinisms, religious values and gender stereotypes into four possible subthemes identified for care: survival instinct; female gaze; nursing; relationship with a higher being. S17: “[…] For this specific scenario, women stand out as recipients of care teaching-learning, highlighting that this social function is practically exclusive to them due to their ability to procreate”.^(30)^	S23: It questions the reproduction of gender stereotypes in the maternal behaviors of nursing professors at universities, trapped in a veiled moral obligation of care. The reinforcement of gender roles is problematized based on feminist epistemology. S23: “Hegemonic femininity in nursing can be identified as the imposition of behavioral norms associated with the feminine […] often resulting in horizontal oppression. […] The imposition of ostensibly feminine behaviors can arouse ‘maternal’ expectations of women in the workplace”.^(7)^
S18: Quantitative correlational study that reinforces, without question, the inferiority of female nursing students in relation to men, presumably more prone to critical thinking. S18: “In this study, students’ femininity was positively associated with caring behavior. There was no significant correlation, however, between femininity and critical thinking […] those who reported greater masculinity displayed greater caring behavior and critical thinking than their fewer male counterparts.”^(31)^	S25: Nursing care is conceived as a political practice influenced by participation in social movements to combat inequalities in the profession. S25: “In the context of health services, some oppressive, silencing practices that blame, judge and victimize, especially other women, and romanticized family care are narrated as recurring […] Due to these characteristics, the care offered is understood as inadequate and harmful”.^(37)^
S19: Two factors were extracted that summarize undergraduate students’ conceptions of care: the first, psychosocial, the second, technical-professional. Psychosocial is associated with women, without statistical significance, with no questioning of gender stereotypes. S19: “Regarding the influence of gender, among the women surveyed, five of the six dimensions most identified with care were related to the psychosocial aspect, and this may lead us to think that women are more concerned with relational and contextual aspects.”^(32)^	
S20: A descriptive cross-sectional study that assumes care as a feminine attribute. Therefore, male nursing students would have difficulties in clinical practice. This association between female care and a negative image of nursing is ratified as alleged essentialized self-evidence. S20: “Several researchers have found that male nursing students encounter more challenges in the clinical setting than female students […], primarily because nursing combines professional and feminine values of caring”.^(34)^	
S24: The research reinforces gender violence in the profession’s scientific discourse, with stereotypes full of moralizations of care, seen as a gesture of altruism, love and affection. S24: “The way of caring for others is revealed when one cares about putting themselves in the other’s shoes, giving love and affection”.^(36)^	

Source: Own preparation.

Of the 25 studies included, qualitative research (n = 15; 60%) was the predominant method, with the presence of reflections or theoretical essays (n = 4; 16%), narrative or systematic literature reviews (n = 3; 12%) as well as quantitative studies (n = 3; 12%). As for publication locations, they were concentrated in journals from Brazil (n = 18; 48%), England (n = 3; 12%), Colombia (n = 2; 8%), Spain (n = 2; 8%) and from Mexico (n = 2; 8%). The rest (n = 4; 16%) were distributed between Cuba, Scotland, USA and Canada. Among global regions, studies were concentrated in the Americas (n = 19; 76%) and Europe (n = 6; 24%). Regarding the year of publication, a greater number of articles occurred in from 2014 to 2019 (n = 14; 56%), followed by intervals from 1996 to 2013 (n = 6; 24%) and from 2020 to 2022 (n = 5; 20%). In the temporal distribution of articles between stereotype criticism (n = 11; 44%) or reissues (n = 14; 66%), we did not observe noteworthy regularities.

The research included in the review presents the following objects of study (Chart 2): i – Experiences, practices, conceptions, rationality and/or learning of men in nursing (S1; S2; S18; S20); ii – Concepts of nursing or caring for students, nurses and/or caregivers of both sexes (S12; S14; S17; S19; S24); iii – Tasks and/or moralizing ethics of care as feminine/maternal (S5; S10; S11; S15; S16); iv – Theories about the political dimension, gender perspective and/or sexuality in nursing care (S3; S6; S7; S23; S25); v – Analysis of gender perspective in teaching, nursing research, care, choice of profession and/or inequalities in care work (S4; S8; S9; S13; S21; S22). The subjects participating in the investigations included nursing students (n = 968; 81.4%), distributed among women (n = 514; 53%), men (n = 390; 40.2%) or without specification (n = 43; 4.4%), female pedagogy, nursing or administration students (n = 21; 2.2%), female nurses (n = 58; 79.4%), male nurses (n = 15; 20, 5%), female caregivers (n = 66; 71.7%), male caregivers (n = 26; 28.2%) and low-income women (n = 56; 4.7%).

Although most articles uncritically reproduce the “natural caregiver” stereotype (n=14; 56%), epistemic resistance (n = 11; 44%) criticize gender inequalities in the profession, constituting an explicit counterpoint. The mapped evidence was grouped into six interrelated categories, three for reissue and three for natural caregiver stereotype criticism. The categories, with their respective references, are as follows: reissue: a) care as essentially feminine (S1; S2; S10; S12; S15; S17; S18; S19; S20)^(15,16,23,25,28,30,31,32,34)^; b) care as a calling and service of love (S5; S11; S24)^(19,24,36)^; c) safety/erasure of gender inequalities (S14; S16)^(27,29)^; criticism: d) “inadequate and harmful” care (S3; S4; S23; S25)^(7,17,18,37)^; e) neutralization of gender and bodies (S6; S8; S9)^(20,21,22)^; f) reporting gender oppression in care work (S7; S13; S22)^(1,26,35)^.

Among the results of studies that reissue gender stereotypes in nursing (n = 14; 66%), we found ratifications of binary conceptions of gender (n = 5; 20%), which reproduce stereotypes that label emotions as a feminine attribute and reason as masculine (S1; S2; S18; S19; E-20). This group includes quantitative studies that analyze men’s experiences in nursing (S1; S2; S20), with the hegemony of male researchers in authorship. Other investigations reproduced concepts of care as synonymous with love, altruism, femininity or as a procreative function, with evident gender violence against women (S5; S11; S12; S14; S17). The “natural caregiver” essentialization as a nurse, mother and woman is strongly manifested in studies that set out to reflect on care work (S10; S14; S15; S16). Studies that confirm gender stereotypes in nursing make the sections by population studied invisible in the analyzes (students, nursing professionals or caregivers, women). In other words, they tend to treat subjects as a homogeneous block, without major differentiations of gender, social class, race or generation when discussing the results.

In turn, the articles that criticize gender stereotypes in nursing (n = 11; 44%), although based on similar objects of study and subjects, differ by the problematization of the analyzes carried out. Some of these, especially theoretical and qualitative, assume the centrality of the political in the profession’s concept and practice (S3; S7; S25). Others denounce naturalization of care as essentially feminine, revealing discursive injustices (S4; S8; S9; S13; S21). Nursing as a social practice, as well as questions about sexism and gender inequalities, are highlighted in part of critical research (S6; S21; S23). In qualitative research on articles classified as critical, gender aspects in the studied population are prioritized in the analyzes carried out (S4; S6; S13; S21; S25).

## DISCUSSION

The concentration of articles published in journals from the global region of the Americas (n = 19; 76%) can be explained by the exploratory phase search engines (BDENF via VHL) and the preponderance of studies from South America in SciELO. Despite this limitation, the preference for clinical and epidemiological research in health journals, which are not used to epistemic discussions of care, may have contributed to the scarce studies from other global regions, deserving further investigation.

Given the complex characteristics of care, the qualitative methodologies present in the articles are suitable for studying the object, as they allow for the deepening of singularities. However, if we consider the interdisciplinarity of feminist epistemology to the criticism of gender stereotypes^(2,3,4,12)^, the few theoretical reflections produced seem to compromise nursing science’s critical potential. As we know, theoretical reflections are in better dialogue with the approaches of the human and social sciences, as they come from these fields. In this context, we highlight the interdisciplinary capillarity in studies on gender, given the training of researchers of critical articles, almost all of whom have a doctoral, post-doctoral or research in gender, human or social sciences (S3; S4; S6; S7; S8; S9; S13; S21; S22; S23; S25),^(7,17, 18,20,1,21,22,26,33,35,37)^ which had an impact on the authors’ studies. Thus, the coincidence between critical articles and the interdisciplinarity in the researchers’ titles indicate the need to expand nurse training for critical analysis of gender issues in the profession, in dialogue with feminist epistemology.

Gender as a category of analysis that problematizes the essentialism of “Woman” (capital letter as a denunciation of totalitarianism and semantic rigidity) was the great contribution of feminist epistemology to the sciences^(2,3,4,12)^. Consequently, the gender perspective^(11)^ introduced a relevant questioning approach to the discursive results of reviewed investigations. We noted this difference in studies that assumed the centrality of the political in nursing conceptions and practices (S3; S7; S25), in those that denounced naturalization of care as feminine (S4; S8; S9; S13; S21) or in those who questioned sexism and gender inequities in the profession (S6; S21; S23).

In turn, the hegemony of positivism, technicalism and productivism in health professions^(38)^ – maintainer of the biomedical, patriarchal, market and socially unfair model for women^(2,3,4,5)^ – feeds back the insufficiency of critical theorizing in the area. Added to this is the almost non-existent space dedicated to theoretical reflections in health journals, pressured by the utilitarianism of science, which discourages research with a reflective and political bent on care. This positivist scenario, linked to the historicity of nursing immersed in sexist, racist and elitist ideologies^(39)^, conforms to the critical insufficiency of articles that reproduced gender stereotypes. More than half of these studies reaffirm care as “essentially feminine”, without any filter regarding its oppressive nature for us, women^(2,3,4,12)^.

The results of uncritical studies in nursing comprise conceptions restricted to gender binarism (S1; S2; S18; S19; E-20), full of moralizing stereotypes about care, resulting in violence for women, whether nurses or not (S5; S11; S12; S14; S17). Inequalities of care work^(2,3,4)^ are also made invisible in the prejudice of “natural caregivers”, sometimes associated with an ideological lack of distinction between nurse, caregiver and mother (S10; S14; S15; S16). As a common trait to biomedical positivism^(38)^, we highlight the total erasure of differences of gender, class, race or generation in the population segment investigated, a characteristic of the alleged scientific neutrality, generating inequities. Furthermore, we observed rigid demarcation in gender roles, with women’s subordination, in the articles that proposed to investigate the presence of men in nursing (S1; S2; S20), mostly with male authorship. This finding reveals the supposed exemption of positive science and researchers (notably men), outlining sexist views and self-reference of the profession’s stereotypical discourses. To put it more clearly: male researchers tended to investigate themselves in nursing, reproducing their sexism. It would be redundant to say that injustices of class, race, gender and generation shape the historicity of nursing; therefore, stigmatized speeches only insult us^(11,38,39)^.

We verified reinforcement of gender stereotype in nursing discourses in the statements that “aspects of care came naturally”^15^ for “female colleagues” (S1)^(15)^, because “a gender link between care and femininity”^(16)^ is “increasingly […] necessary to transmit care” (S2)^(16)^. In other voices, we read that “the feminine way of being”^(23)^ predisposes to “sensitivity and personal involvement with the suffering of others” (S10)^(23)^, since women “have learned the role of caregiver […] and improved their skills by participating in the daily care of their children” (S15)^(28)^. In these studies, “Women” are seen as “recipients of care teaching-learning”,^(30)^ understood as a “social function” that would be “practically exclusive due to their ability to procreate”^(30)^ (S17).

In categories a) care as essentially feminine and b) care as a calling and service of love, we observed the entrenchment of gender roles that typify women as sensitive, emotional and with little use of reason, reissued in the researchers’ speeches. These nurses argue, without filters, that “women are more concerned with relational aspects” (S19)^(32)^. The same reproduction of gender stereotypes can be seen in research with undergraduate nursing students, which concludes that “femininity of students”^(31)^ is “associated with caring behavior”,^(31)^ whereas “greater masculinity”^(31)^ relates to “critical thinking” (S18)^(31)^. Intrinsically intertwined with these distortions, the view of care as a “call to service” of love complements the discriminatory tone of the speeches that attack us, without any filter, expressed by the authors. Finally, in category c) harmlessness of inequalities, the researchers erased injustices related to gender, seen as a synonym for sex, associated with the uncritical exaltation of a “superwoman” (S16)^(29)^, as if it were an immutable and biologically determined phenomenon.

We highlighted an excerpt that summarizes the “natural caregiver” stereotype without parsimony, reinforcing symbolic violence against us, women nurses, which deserves deep reflection. We referred to the study that considers nursing not as a job, a social practice or a historical profession, but “a quality that some people possess innately”,^(25)^ which leads to a “special predisposition, called vocation, to help others”.^(25)^ Nursing, colleagues reiterate without any reservations, would be “an activity linked to women and related to motherhood”,^(25)^ which “provides the motivation and impulse necessary to provide care” (S12)^(25)^.

Conceiving nursing as a feminine attribute, in an immutable essentialist view, as well as care as an “impulse” of motherhood, it only encourages sexist practices and discourses considered “natural” – when they are socially produced to deepen gender oppression in the profession. The study authors do not make it clear what they understand by “impulse” (S12)^(25)^, perhaps because they assume the essentially caring nature of women is “given” and “self-evident”, therefore exempt from any questioning. Nor do they discuss the harmful repercussions for us, nurses, if we consider care, rather than a social practice situated in power relations – therefore in strategic and flexible situations of domains subject to change through correlations of forces^(17)^ – a kind of Freudian drive, i.e., an uncontrollable “psychological and endosomal representation”^(40)^. In short, we read in this research that the “innate quality” of nursing (and not the work!) would predispose us to helping others and would be present in us, procreating object-women, as a force of nature that we cannot fight, counter, resist or critically deny, only passively accept.

Nothing is more contrary to the social and political achievements we need than discourses that germinate and incubate gender inequalities regarding “natural caregivers” in our bodies^(11)^. The mistakes, inequities and damage caused by this type of science, surprisingly produced by the nursing elite, are pressing. In striking contrast to the idealizations of the profession as “a call to serve” (S5)^(19)^, we should briefly remember the terrible working conditions, violence, discrimination, low wages, the absence of a minimum wage or the political fragility of the category in labor struggles, among other injustices contextualized in introduction^(1–7,9)^. Although these statements are explained structurally in the profession’s historicity, permeated by conservative, sexist and racist ideologies that vituperate us^(7,10,17,37)^, they do not determine the entirety of the profession’s scientific discourses. Furthermore, they highlight a majority who do not perceive themselves as trapped in their own discourse, nor do they identify the hostile repercussions for us, women, in uncritical dissemination of “natural caregiver” stereotype in nursing.

As resistance in the scientific field of the profession, other studies problematized inequalities arising from gender stereotypes, grouped into the following categories: d) “inadequate and harmful” care; e) neutralization of gender and bodies; and f) reporting gender oppression in care work. Representatives of these classifications expressed divergent thinking supported by feminist epistemology, as we said above. We borrowed the expression “inadequate and harmful” care (S25)^(37)^, mentioned in one of the articles, to name the first critical category. In their arguments, the researchers point to care as a social and political practice (S3)^(17)^; question the hierarchical relationships established between men and women in care (S4)^(18)^; denounce the “imposition of norms associated with the feminine” (S23)^(7)^ and naturalization of the “relationship between care and feminine action”, as well as the “maternal expectations of women in the workplace”.^(7)^ Critical studies also revealed the narratives oppressors who “blame, judge and victimize” (S25)^(37)^ women and care.

In turn, in articles about the neutralization of gender and bodies, the second critical category, scientists contested sexual interdiction and denial in nurses’ erotic bodies (S6)^(20)^; problematized the “social order of gender” (S8)^(21)^ from the patriarchal context; as well as contesting the restricted conception of gender in nursing studies, most of which focus on “invariable and fixed constructs” (S9)^(22)^. In other words, researchers criticize the conception of gender that prevails in nursing science, which remains linked exclusively to the restricted conception of women or erases hierarchical differences (S9)^(22)^. In the third critical category, the articles denounce gender oppression in care work, falsely justified in the “natural caregiver” stereotype (A7)^(1)^. Nursing care scholars defend changes in the representations of women’s work as an “extension of domestic functions” (S14)^(27)^, since the “break with the roles socially designated for women” (S22)^(35)^ contributes to a fairer distribution of care work.

In fact, one of the relevant discussions in feminist epistemology argues that the relationship between care and capitalism passes through the reproductive dimension of work, whose responsibility falls unequally on women (or “servants of capital”)^(2)^. In other words, in a context of global economy, productive work, which generates accumulation through exchange values, inseparably needs the reproductive dimension, that which produces a healthy workforce to be exploited, forged in life-sustaining activities (domestic tasks, health care, child care, elder care, well-being, etc.). In other words, in the complexity of productive care relations, capitalism cannot survive without the work unequally attributed to women. In this area, the subversion of the patriarchal logic of distribution of care tasks can make the relationship between genders more equitable, having repercussions on democracy^(2–4)^.

Similar to health, given the quantitative majority of nursing workers, the provision of services depends viscerally on nurses and technicians. Despite this majority support, the discriminatory bias that we voluntarily proclaim makes our bodies docile, dulls our reflections, silences our voices and undermines our political strength to change the unworthy working conditions. Nevertheless, the contributions of this and other studies in identifying critical resistance within the epistemic field of nursing may tip the balance in our favor.

In this context, strategies for confronting gender stereotypes in nursing involve greater articulation between research, education, political organization and practice. In the context of training nurses and staff, it is urgent to include a gender perspective in the critical problematization of endogenous discourses that imprison us in symbolic oppression, as they only increase violence, salary devaluation and unworthy working conditions. Feminist epistemology constitutes a necessary approach to disciplinary and extracurricular content, especially those that dialogue with historicity, care and professional practice. Furthermore, nursing research and science, linked to teaching and extension, can better dialogue with feminist frameworks in the production of critical thoughts and political engagements in nursing. Within the scope of class associations, we need to problematize, in depth, in the various discussion forums, how unfair, cruel and oppressive the ideological discourses we reproduce are, expanding our power to confront inequities. In the short term, the results of this article can support problematizing discussions about care and gender stereotypes in nursing scientific research in different learning scenarios among nursing students, professors and nurses.

### Study Limitations

Since we did not contact the authors of articles not available on the internet, some important references may have been removed from the study. The exclusion of gray literature may have reduced the number of critical studies of gender stereotypes in nursing, present in master’s dissertations and doctoral theses.

## CONCLUSION

Nursing scientific research on care, for the most part, reproduces gender stereotypes and idealizations that reinforce the oppression of women in the profession. In these studies, discourses prevail that crystallize a “natural caregiver” and point out care linked to the feminine, as if it were “a calling and service of love” – never a social relationship of powers in dispute, disruptive.

In contrast, critical resistance from nurse scientists denounces female naturalization of care as “inadequate and harmful”, for perpetuating gender oppression. These studies are based on feminist epistemology and use gender as an analytical dimension that questions binaries, prohibitions on the body, false neutralities and the erasure of sexism. In line with these studies, we ask how much these questions are part of our scientific work to impact the critical training of nurses and technicians, or what would happen if we accumulated discursive practices to confront marketing, misogynistic, biomedical and patriarchal powers behind every “kind” care, i.e., that false (feminine) “impulse” to subserve.

Given the centrality of care for nursing, the findings of this review indicate the need to expand self-criticism regarding the profession’s scientific discourses, in order to reveal sexist patterns that violate us in an endogenous, invisible and uncritical manner. The implications of this study for nursing research point to a gap in scientific studies on care from a gender perspective, with incipient power of criticism from feminist epistemology. Within the scope of nursing practices, the reissue of gender stereotypes in nurse researchers’ discourse, in addition to maintaining unworthy working conditions, hinders the achievement of rights, autonomy and professional development.
