# Supplementary material for: Analysis of care and gender stereotypes in nursing scientific research: a scoping review
Source: Rev Esc Enferm USP. 2024 Sep 9;58:e20240066. doi: 10.1590/1980-220X-REEUSP-2024-0066en (PMC11391781; doi:10.1590/1980-220X-REEUSP-2024-0066en)
Supplement: Translation

# Translation: Análise do cuidado e estereótipos de gênero na produção científica da enfermagem: revisão de escopo

**DOI:** 10.1590/1980-220X-REEUSP-2024-0066pt

**Authors:** Gabriela Duarte Almeida Mundim, Maria Raquel Gomes Maia Pires, Maria Verônica Sousa Torres, Aline Oliveira Silveira

**Affiliations:** 1 Universidade de Brasília, Centro de Estudos Avançados Multidisciplinares, Brasília, DF, Brasil.; 2 Universidade de Brasília, Faculdade de Ciências da Saúde, Departamento de Enfermagem, Brasília, DF, Brasil.

**Keywords:** Cuidados de Enfermagem, Identidade de Gênero, Enfermagem, Feminismo

## Abstract

**Objetivo:**

Mapear evidencias sobre o cuidado e os estereótipos de gênero na produção científica de enfermagem.

**Método:**

Revisão de escopo desenvolvida sob o referencial do *Joanna Briggs Institute* com análise da perspectiva de gênero nas abordagens de cuidado. As buscas foram realizadas em 31 de janeiro de 2023, nas bases: SciELO, SCOPUS, CINAHL, PUBMED, BDENF.

**Resultados:**

Dos 3.743 estudos localizados, 25 foram incluídos. As evidências foram agrupadas nas categorias: cuidado essencialmente feminino (n = 9; 36%); chamado e serviço do amor (n = 3; 12%); apagamento das desigualdades de gênero (n = 2; 8%); cuidado “inadequado e danoso” (n = 5; 20%); neutralização do gênero e dos corpos (n = 3; 12%); e denúncia das opressões no trabalho do cuidado (n = 3; 12%).

**Conclusão:**

A maioria da produção científica sobre o cuidado reproduz estereótipos de gênero que reforçam as opressões sobre as mulheres na enfermagem. Em contraponto, as resistências denunciam a naturalização do cuidado como “inadequado e danoso”, por perpetuarem as opressões de gênero no trabalho do cuidado.

## INTRODUÇÃO

O estereótipo da “cuidadora natural”, marcado por questões de gênero, classe, raça e geração, cristaliza a imagem da mulher como supostamente “vocacionada” a cuidar em vista de sua natureza feminina, repercutindo em desigualdades de gênero, sobretudo na enfermagem. O rótulo da cuidadora natural reforça a ideia de que o trabalho do cuidado seria destinado para as mulheres, condicionado fixamente pela natureza feminina atrelada ao determinismo biológico, à revelia de serem construções sociais. Nessa ótica, se tomarmos o cuidado como elemento central para a concretização da democracia, vemos as repercussões injustas desse estereótipo, entre elas: a precarização das relações de trabalho; a dificuldade de acesso aos espaços políticos; a tripla jornada de trabalho; a baixa remuneração; o incipiente reconhecimento social; bem como a expropriação do tempo e energia das mulheres, questões que implicam em maiores injustiças^(1-3)^.

É bem debatido na literatura que o trabalho do cuidado – de crianças, idosos, doentes e do lar – majoritariamente realizado por mulheres, enfermeiras ou não, é socialmente desvalorizado, mal remunerado (ou não remunerado) e precarizado. A concepção mais aceita de trabalho do cuidado, oriunda da sociologia das emoções, diz respeito à atenção constante e intensa que uma pessoa faz para o bem-estar de outra. A enfermagem, como profissão inserida no trabalho do cuidado, igualmente se depara com as injustas condições laborais, especialmente na área assistencial. Recente pesquisa sobre o perfil profissional no Brasil aponta susceptibilidades no mercado de trabalho, tais como: desvalorização com baixos salários; precarização dos vínculos; multiempregos; dificuldades de alocação; insegurança e violência no ambiente de trabalho, entre outras^(4,5)^.

Na enfermagem, os estereótipos marcam o imaginário e a realidade da profissão desde a origem, aprisionando-a em adjetivações fixas que aprofundam as desigualdades nas condições de trabalho. Os estereótipos podem ser compreendidos como uma visão preconceituosa e generalizante de características que grupos e indivíduos possuem ou de atributos que a sociedade espera que tenham. Estereotipar consiste em ignorar as características singulares de uma pessoa e tratá-la como um molde. Estudos de revisão identificam estereótipos de gênero nas visões da sociedade sobre a enfermagem, entre eles: para as enfermeiras, presumível incompetência técnica, fraco nível acadêmico e profissional, incipiente autonomia e hipersexualização; para os enfermeiros, o questionamento da masculinidade, faces da mesma injustiça^(1,6)^.

Entrementes, no presente estudo, problematizamos o quanto as visões preconceituosas sobre a profissão são retroalimentadas por nossos discursos científicos. A esse respeito, são emblemáticas as produções que constatam a reedição da “cuidadora natural” nas produções na enfermagem. Os motivos da escolha profissional, por exemplo, continuam marcados por sexismos, conservadorismos e idealizações de perfectibilidades inatingíveis, centradas num altruísmo santificado do “ser enfermeira”. Por seu turno, os homens da profissão demarcam suas escolhas com base em objetivos racionais, como possibilidade de emprego e liderança na categoria, demarcando bem as desigualdades de gênero dentro da profissão. Noutro estudo, a sexualização das enfermeiras, a liderança masculina, a fragilidade emocional da mulher e o cuidado como atributo feminino foram interpretados como parte da “visão da sociedade sobre a profissão”, com ausência de reflexão acerca dos discursos científicos ideologizados na enfermagem. Em consequência, constatamos um abismo entre o que a enfermagem afirma e o cotidiano da profissão, numa contradição endógena^(7,8)^.

Num contraponto crítico, a pandemia de covid-19 exacerbou o quanto os epítetos de enfermeiras “anjos” ou “heróis” não correspondem à dura realidade e à elevada mortalidade no segmento profissional. Uma pesquisa que investigou as percepções dessas narrativas entre profissionais de enfermagem concluiu pela ciência do quão distante as idealizações estão do cotidiano de trabalho das(os) entrevistadas(os). No Brasil, homenagens sentimentais da mídia durante a pandemia não se traduziram na defesa do piso salarial para a enfermagem, que até o momento persiste na luta. Portanto, passa da hora de refletirmos sobre o quanto os discursos que reeditamos nas produções científicas contribuem para tamanha fragilidade política^(9)^.

Esse cenário reitera a importância de investigarmos a naturalização dos estereótipos de gênero, compreendendo-os como manifestações discursivas de intrincadas relações de poderes capazes de limitar rigidamente a prática social e a produção científica da enfermagem a partir de um suposto determinismo social. As repercussões assimétricas da naturalização do cuidado – como caráter estruturante de desigualdades de gênero que interpela às mulheres aptidões pautadas em uma pretensa linearidade causal – justificam a necessidade de ampliar a produção crítica acerca do enfrentamento da naturalização do cuidado como uma condição feminina.

Para análise dos estereótipos de gênero, realizamos anteriormente um estudo teórico sobre a epistemologia feminista e cuidado^(10)^, com delimitação das seguintes dimensões à crítica da “cuidadora natural” na ciência da enfermagem: Gênero – categoria de contestação para qualquer sentido binário de homem/mulher restrito ao sexo biológico que, em contraponto, considera as relações de poderes produzidas nos atos performáticos e discursivos sobre sexo, sexualidade, desejo e gênero na conformação da normatividade heterossexual discriminatória^(11)^; Trabalho do cuidado – tudo que fazemos para o bem-estar de alguém ou alguma coisa, seja na esfera reprodutiva ou produtiva da vida^(2)^; “Cuidadora natural” – destinação exclusiva, desigual e injusta da mulher às tarefas do cuidado, em vista de uma suposta e imutável natureza “feminina”^(1)^. O uso do termo “cuidadora natural”, nesse estudo, sintetiza os muitos estereótipos de gênero a ele atrelados no âmbito da enfermagem.

A partir desses pressupostos, a questão norteadora do artigo é esta: como se apresentam as evidências sobre as abordagens do cuidado na produção científica da enfermagem, no que se refere aos estereótipos de gênero? O estudo se justifica pela centralidade do cuidado para a prática da enfermagem e as poucas revisões de escopo que analisam a perspectiva de gênero nessas produções. Com base nessa premissa, o objetivo é mapear as evidências sobre o cuidado e os estereótipos de gênero na produção científica de enfermagem.

## MÉTODO

### Tipo De Estudo

Trata-se de uma revisão de escopo (*scoping review*) com análise das abordagens do cuidado na produção científica da enfermagem, na perspectiva do gênero. Esse tipo de revisão tem como finalidade identificar conceitos-chave e lacunas de conhecimento passíveis de aprofundamento em estudos futuros, a partir da síntese de evidências presentes na literatura^(12)^.

Procedemos a revisão de escopo sobre as concepções de cuidado na produção científica da enfermagem seguindo o método recomendado pelo *Joanna Briggs Institute* (JBI)^(12,13)^, com as etapas: 1 – identificação da questão a partir do mnemônico PCC: P (População/*Population*); C (Conceito/*Concept*); C (Contexto/*Context*); 2 – critérios de inclusão; 3 – estratégias de pesquisa em duas fases; 4 – extração dos dados com análise da concepção de cuidado na perspectiva de gênero; 5 – sistematização e apresentação dos resultados. Foram utilizadas ainda as recomendações do *checklist Prisma Statement 2020* (Primas-ScR)^(14)^. O protocolo utilizado no estudo foi elaborado e registrado no *Open Science Framework*, sob o *link*: https://osf.io/xv3ph/.

### Questão de Pesquisa

Para a construção da questão de pesquisa, utilizamos o mnemônico PCC: P – enfermagem; C – abordagens do cuidado; C –– estereótipo de gênero; com delimitação da pergunta: como se apresentam as evidências sobre as abordagens do cuidado na produção científica da enfermagem, no que se refere aos estereótipos de gênero?

### Critérios de Inclusão e de Exclusão

Adotamos os seguintes critérios de inclusão: artigos de periódicos científicos, disponibilizados na íntegra, que abordem o cuidado como objeto de reflexão na enfermagem; produções publicadas por enfermeiras e/ou em revistas de enfermagem. Foram excluídos estudos que não contemplavam o cuidado como objeto de discussão.

### Estratégia de Pesquisa

Em vista de a quantidade de produções nas bases de dados investigadas ter sido suficiente para a análise do estereótipo da “cuidadora natural”, optamos por não incluir literatura cinzenta no escopo de busca. Por rigor metodológico, realizamos uma fase exploratória com a inclusão de palavras-chave em português, inglês e francês para averiguar a pertinência dos descritores, das bibliotecas virtuais da enfermagem e das bases de dados. Nessa fase, a busca foi delimitada ao período de 2020 a 2021. Em seguida, na fase de aperfeiçoamento, ampliamos o processo de busca, modificamos os descritores, incluímos apenas os termos em inglês e ajustamos as bases de dados para ampliar progressivamente o processo investigativo. Nessa segunda fase, não estabelecemos limites quanto ao período de publicação ou idioma, pois pretendíamos investigar o escopo dos estudos sobre concepções de cuidado na enfermagem^(12,13)^.

Na identificação de artigos relevantes à temática, pesquisamos nas seguintes bases e/ou bibliotecas: SciELO, SCOPUS, CINAHL, PUBMED, BDENF (Via BVS). Além disso, fizemos a busca manual e reversa nas referências bibliográficas dos artigos identificados. Quanto aos descritores, consideramos os preconizados da *Medical Subject Headings* (MESH) da *National Library of Medicine* (NLM) dos Estados Unidos, bem como os Descritores em Ciências da Saúde (DeCS). Para ampliar o retorno, os operadores booleanos foram utilizados desta forma: i – fase exploratória: (Enfermagem OR Nursing OR Soins Infirmiéres OR Enferm*) AND (Cuidado OR Care OR Soins) AND (Gênero OR Gender OR Genero); ii – fase de aperfeiçoamento: (care OR practice) AND (gender) AND (nursing) AND (research OR studie). O *string* de busca, realizado em 31 de janeiro de 2023, está descrito no Quadro 1.

**Quadro 1:** *String* de busca por bases de dados e fase da revisão – Brasília, DF, 2023.

Base de dados	String de busca	
	Exploratória	Aperfeiçoamento
SciELO	(ab:((Enfermagem OR Nursing OR Soins Infirmiéres OR Enferm*) AND (Cuidado OR Care OR Soins) AND (Gênero OR Gender OR Genero)))	(ab:((care OR practice) AND (gender) AND (nursing) AND (research OR studies)))
BDENF (Via BVS) na Exploratória PUBMED no aperfeiçoamento	((Enfermagem OR Nursing OR Soins Infirmiéres OR Enferm*)) AND ((Cuidado OR Care OR Soins)) AND ((Gênero OR Gender OR Genero)) Limite de busca: título, resumo, assunto.	((care[Title/Abstract] OR practice[Title/Abstract]) AND (gender[Title/Abstract]) AND (nursing[Title/Abstract]) AND (research[Title/Abstract] OR studie[Title/Abstract]))
SCOPUS	(TITLE-ABS-KEY (enfermagem OR enfermagem OR soins AND infirmiéres OR enferm*) AND TITLE-ABS-KEY (cuidado OR care OR soins) AND TITLE-ABS-KEY (gênero OR gender OR genero))	(TITLE-ABS-KEY (care OR practice) AND TITLE-ABS-KEY (gender) AND TITLE-ABS-KEY (nursing) AND TITLE-ABS-KEY (research OR studie))
CINAHL	AB (Enfermagem OR Nursing OR Soins Infirmiéres OR Enferm*) AND AB (Cuidado OR Care OR Soins) AND AB (Gênero OR Gender OR Genero)	AB (care OR practice) AND AB gender AND AB nursing AND AB (research OR studie)

Fonte: Elaboração própria.

### Extração dos Resultados

Os artigos foram pré-selecionados a partir dos títulos e resumos, sendo realizada, em seguida, a leitura na íntegra das produções. Como recomendação da técnica, a triagem em duas etapas (leitura dos títulos e resumos; leitura na íntegra), a extração de dados e a análise dos resultados foram realizadas por duas avaliadoras de forma independente. As divergências foram decididas por uma terceira^(12,13)^. Recorremos aos *softwares* Zotero® para gerenciamento de referências e Rayyan® para tomada de decisão na fase de triagem. Na extração dos resultados, o programa Microsoft Excel possibilitou a organização das produções por ano, título, autoria, local de publicação, idioma, metodologia, objetivo e concepção de cuidado.

Para a análise do cuidado na perspectiva de gênero, adaptamos o instrumento de extração de dados recomendado pelo JBI^(13)^ com a inclusão das seguintes questões, elaboradas a partir das dimensões gênero^(11)^, trabalho do cuidado^(2)^ e estereótipo da “cuidadora natural”^(1)^: 1 – O artigo aborda a questão de gênero em perspectiva feminista e contesta a relação binária? 2 – As abordagens de cuidado discutem as relações de poder e de gênero intrínsecas ao trabalho do cuidado? 3 – A produção científica reflete sobre as repercussões das desigualdades de gênero no trabalho da enfermagem? 4 – O artigo reedita valores morais, discursos ou práticas de que as mulheres “nasceram para cuidar”? Na crítica do estereótipo, consideramos as produções que contemplam afirmativamente pelo menos uma das questões de 1 a 3. Para a reedição, classificamos aqueles com ausência de discussão das dimensões de gênero e trabalho do cuidado, com reafirmação do estereótipo da cuidadora natural. Aqueles que descreveram a problemática da desigualdade, sem análise ou posicionamento, consideramos reedição.

### Análise e Apresentação dos Resultados

Na sistematização dos resultados, realizamos a análise de conteúdo dos artigos com a extração das respectivas categorias empíricas. Para isso, inicialmente procedemos a leitura na íntegra do material flutuante, com destaque de trechos considerados relevantes para a investigação, no que se refere à reedição ou à crítica dos estereótipos de gênero na produção científica da enfermagem. Em seguida, produzimos planilhas no Microsoft Excel com a classificação dos 25 artigos segundo respostas às quatro perguntas norteadoras elaboradas (1 – O artigo aborda a questão de gênero em perspectiva feminista e contesta a relação binária? 2 – As abordagens de cuidado discutem as relações de poder e de gênero intrínsecas ao trabalho do cuidado? 3 – A produção científica reflete sobre as repercussões das desigualdades de gênero no trabalho da enfermagem? 4 – O artigo reedita valores morais, discursos ou práticas de que as mulheres “nasceram para cuidar”?). Cada artigo foi analisado conforme reedição ou crítica dos estereótipos, com extração de trechos representativos para justificar cada resposta às perguntas norteadoras. A partir dessa primeira tipificação, realizamos a extração de seis categorias empíricas do conteúdo selecionado, sendo três representativas de reedição e três que informam a crítica dos estereótipos de gênero. As categorias empíricas extraídas dos artigos foram as seguintes. Reedição: a) cuidado essencialmente feminino; b) chamado e serviço do amor; c) apagamento das desigualdades de gênero. Crítica: d) cuidado “inadequado e danoso”; e) neutralização do gênero e dos corpos; f) denúncia das opressões no trabalho do cuidado. Para efeitos de maior visualização objetiva do mapeamento e discussão das evidências, optamos pela classificação em números e percentuais da quantidade de artigos agrupados em cada uma dessas categorias. Na apresentação dos resultados, utilizamos o fluxograma de PRISMA, um quadro com a caracterização dos 25 estudos e outro com a exemplificação dos trechos representativos da reedição ou da crítica do estereótipo de gênero, conforme o caso. No quadro 2, referente a descrição dos 25 estudos, estabelecemos códigos de identificação (ID) para cada uma das produções incluídas, numeradas de E1 a E25.

**Quadro 2:** Caracterização dos artigos incluídos na revisão de escopo por título resumido, local de publicação, objetivo, participantes e metodologia – Brasília, DF, 2024.

ID	Título	Local e ano	Objetivo	Participantes	Metodologia
E1	Learning to care: gender issues for male nursing students^(15)^	Canadá, 1996	revelar similaridades e diferenças nas experiências vividas por estudantes de enfermagem homens	20 estudantes de enfermagem do sexo masculino	Pesquisa qualitativa
E2	The concept of care in male nurse work^(16)^	Inglaterra, 2001	analisar as experiências dos participantes e compará-las com a literatura sobre o conceito de cuidado na prática de enfermagem	8 enfermeiros do sexo masculino	Pesquisa qualitativa
E3	Politicidade do cuidado como referência emancipatória para a enfermagem^(17)^	Brasil, 2005	teorizar a politicidade do cuidado e apontar dinâmicas disruptivas para a enfermagem a partir do triedro do cuidar	Não se aplica	Reflexão teórica
E4	A questão do gênero no ensinar em enfermagem^(18)^	Brasil, 2009	analisar a questão de gênero no ensino do cuidado na formação da enfermeira	21 enfermeiras mulheres, sendo 13 egressas da UEFS e 8 docentes da área de Saúde do Adulto	Pesquisa qualitativa
E5	Nursing care from the perspective of ethics of care and of gender^(19)^	Colômbia, 2013	explorar as dimensões éticas do conceito e da prática do cuidado, em uma perspectiva de gênero	11 profissionais de enfermagem (6 mulheres e 5 homens) que trabalham no Hospital Base em Valdivia, Chile	Pesquisa qualitativa
E6	Sexualidade e a interseção com o cuidado na prática profissional de enfermeiras^(20)^	Brasil, 2013	analisar a interseção entre sexualidade e cuidado de enfermagem enquanto prática social	09 enfermeiras de Barbacena, Minas Gerais	Pesquisa qualitativa
E7	A politicidade do cuidado na crítica aos estereótipos de gênero^(1)^	Brasil, 2016	analisar as desigualdades de gênero entre mulheres brasileiras em Portugal e na enfermagem	Não se aplica	Reflexão teórica
E8	Aportes del enfoque de género en la investigación de cuidadores primário^(21)^	Espanha, 2017	revisar estudos que incorporam o enfoque de gênero no cuidado	Não se aplica	Pesquisa qualitativa
E9	La categoría de género en la investigación y producción de conocimiento en enfermería en Iberoamérica: aportes para el debate^(22)^	México, 2017	visibilizar vieses ou cegueira de gênero na produção de conhecimento da enfermagem ibero-americana	Não se aplica	Revisão narrativa
E10	Ser mãe e enfermeira: questões sobre gênero e a sobreposição de papéis sociais^(23)^	Brasil, 2017	descrever as experiências de mães enfermeiras na conciliação de seus papéis sociais	10 enfermeiras mães	Pesquisa qualitativa
E11	Cuidado ético do outro: contribuições de Edith Stein e Max Scheler^(24)^	Brasil, 2018	analisar a empatia de Edith Stein e a simpatia de Max Scheler para um cuidado ético do outro	Não se aplica	Reflexão teórica
E12	Emergencia del modelo de enfermería transmitido en las universidades españolas: una aproximación analítica a través de la Teoría Fundamentada^(25)^	Brasil, 2018	conhecer o significado do termo enfermagem para as enfermeiras docentes em universidades espanholas	08 enfermeiras(os) docentes de universidades espanholas (6 mulheres e 2 homens)	Pesquisa qualitativa
E13	Entre o Estado, a sociedade e a família: o care das mulheres cuidadoras^(26)^	Brasil, 2018	investigar o care realizado por cuidadoras familiares de idosos dependentes e suas repercussões sociais	45 cuidadoras de idosos (36 mulheres e 9 homens) acompanhados em Serviços de Assistência Domiciliar	Pesquisa qualitativa
E14	Estructura y organización de las representaciones sociales del concepto cuidar^(27)^	México, 2018	analisar a estrutura e organização das representações sociais do conceito de cuidar nos cuidadores	38 cuidadoras(es) de pessoas com doenças crônicas (21 mulheres e 17 homens)	Pesquisa qualitativa
E15	Gender and informal care: different sense and meanings for men and women^(28)^	Brasil, 2018	examinar as tarefas diárias dos cuidados de saúde de mulheres de baixa renda no noroeste de Córdoba	56 mulheres de baixa renda	Pesquisa qualitativa
E16	La cuidadora familiar: sentimiento de obligación naturalizado de la mujer^(29)^	Espanha, 2018	visibilizar a função do cuidado familiar restrita às mulheres como parte de papéis de gênero motivados por um sentimento de obrigação naturalizado	09 mulheres cuidadoras	Pesquisa qualitativa
E17	Meaning of care before starting nursing professional training^(30)^	Cuba, 2018	interpretar o significado do cuidado de estudantes de enfermagem	06 estudantes matriculados no primeiro semestre de enfermagem (4 mulheres e 2 homens)	Pesquisa qualitativa
E18	The effect of gender role orientation on student nurses’ caring behaviour and critical thinking^(31)^	Inglaterra, 2018	explorar o impacto dos papéis de gênero no pensamento crítico e nas práticas de cuidado de estudantes de enfermagem	449 estudantes de enfermagem que tiveram pelo menos um mês de experiência na prática clínica (310 mulheres e 139 homens)	Pesquisa quantitativa
E19	Perception of caring among nursing students: Results from a cross-sectional survey^(32)^	Escócia, 2019	analisar a percepção de cuidado entre estudantes de enfermagem espanhóis	321 estudantes de enfermagem espanhóis (200 mulheres, 88 homens, 33 sem respostas)	Pesquisa quantitativa
E20	Resistência e resignação: narrativas de gênero na escolha da enfermagem^(33)^	Brasil, 2020	analisar narrativas sobre o processo de escolha dos estudos superiores de estudantes mulheres matriculadas nos cursos de Enfermagem e Pedagogia	21 estudantes universitárias mulheres dos cursos de enfermagem, pedagogia e administração de IES privadas em São Paulo	Pesquisa qualitativa
E21	The effect of gender-friendliness barriers on perceived image in nursing and caring behaviour among male nursing students^(34)^	Inglaterra, 2019	examinar as relações entre a imagem da enfermagem, os comportamentos de cuidado e as barreiras de gênero experienciadas por estudantes de enfermagem do sexo masculino	141 estudantes de enfermagem do sexo masculino que obtiveram pelo menos 1 mês de experiência em prática clínica	Pesquisa quantitativa
E22	Burden and Gender inequalities around Informal Care^(35)^	Colômbia, 2020	entender as consequências do cuidado informal para a cuidadora em um debate na perspectiva de gênero	Não se aplica	Revisão narrativa
E23	Paying the Caring Tax: The Detrimental Influences of Gender^(7)^	EUA, 2020	analisar as desigualdades de gênero avindas de imposições morais sobre o cuidado das enfermeiras nos locais de trabalho	Não se aplica	Ensaio teórico
E24	Sentidos do cuidado para acadêmicos de enfermagem^(36)^	Brasil, 2020	identificar os sentidos do cuidado para estudantes de enfermagem	13 acadêmicos de enfermagem do último período de graduação em uma Faculdade de Enfermagem (sem especificação de sexo)	Pesquisa qualitativa
E25	Que não seja aquela enfermagem que pede silêncio^(37)^	Brasil, 2022	analisar os saberes sociopolíticos de enfermeiras em movimentos sociais	6 mulheres-enfermeiras inseridas em movimentos sociais e com representação política	Pesquisa qualitativa

Fonte: Elaboração própria.

### Disponibilidade de Dados

Conforme preconizado pela Ciência Aberta, os dados de pesquisa foram depositados em repositório de acesso público, sob o link: https://doi.org/10.48331/scielodata.VKXGGD.

## RESULTADOS

Retornaram da busca 3.743 estudos, que, com a remoção dos duplicados, resultaram em 2.529. Na primeira etapa de triagem, por meio da leitura do título e do resumo, foram excluídos 2.462 artigos. Na segunda etapa de triagem, 67 artigos foram lidos na íntegra e 42 foram excluídos pelos seguintes motivos: população não era a enfermagem (n = 24); o cuidado não era o conceito abordado (n = 7); e não contextualizavam o estereótipo de gênero (n = 11). A amostra final foi composta por 25 estudos. A Figura 1 apresenta o fluxograma PRISMA^(14)^ da presente revisão.

**Figura 1:** Fluxograma PRISMA de seleção dos estudos.

Fonte: Fluxograma PRISMA de seleção de estudos.^14^

O Quadro 2 apresenta a descrição dos 25 artigos incluídos na presente revisão em relação a identificação do estudo, título, local e ano de publicação, objetivo, participantes e metodologia. O Quadro 3 analisa a reedição ou a crítica presente nas produções quanto ao estereótipo da cuidadora natural, com exemplificação de citação direta extraída dos respectivos artigos. Todas as traduções realizadas nesses quadros são de nossa responsabilidade.

**Quadro 3:** Análise das abordagens do cuidado na produção científica de enfermagem incluída na revisão de escopo em relação ao estereótipo da cuidadora natural, segundo resultado e trecho exemplificador – Brasil, Brasília, DF, 2024.

Reedição do estereótipo da “cuidadora natural”	Crítica do estereótipo da “cuidadora natural”
E1: O estudo reforça concepções de gênero binário restritas a duas formas de cuidar: uma ‘masculina’, pretensamente aprendida pelos estudantes homens; outra ‘feminina’, supostamente natural para as mulheres. E1: “Os estudantes calouros […] admitem que aprenderam aspectos do cuidado que ‘vieram naturalmente’ para as colegas mulheres […] É preciso uma avaliação honesta sobre como as mulheres na enfermagem definiram o cuidado como trabalho feminino […]”.^(15)^	E3: Defende-se a tese da centralidade da dimensão política do cuidado. Argumenta-se em prol de uma nova lógica do cuidar, onde se exercite um auxílio que, sendo poder, tanto subjuga, como emancipa. E3: “A politicidade do cuidado reside na intrínseca ambivalência da ajuda que, sendo poder, tanto domina como liberta fazeres humanos. […] Trata-se de politizar a prática social da enfermagem nos ricos espaços onde se insere, partilhando decisões e ampliando o debate em torno das diferenças.”^(17)^
E2: O modelo conceitual de cuidado proposto ratifica estereótipos de gênero sexistas, quais sejam, um perfil mais emocional para as enfermeiras mulheres, em relação à força física e a virilidade dos enfermeiros homens. E2: “Autores apontam que frequentemente é feita uma vinculação de gênero entre o cuidado e feminilidade e que, cada vez mais, esta é vista como necessário para transmitir o cuidado aos alunos”^(16)^	E4: Evidenciados e criticados nos discursos das enfermeiras docentes estereótipos de gênero relacionados ao cuidado, com direcionamentos para a problematização dessas questões na formação e na prática da enfermagem. E4: “O entendimento das relações estabelecidas entre homens e mulheres que prestam cuidado contribuirá para possíveis rupturas no seio da enfermagem, que sofre um processo de naturalização do seu fazer, encarado como uma extensão das atividades domésticas exercidas por mulheres.”^(18)^
E5: O estudo reproduz ideologias, valores morais e segregações de gênero nos discursos de homens e das mulheres na enfermagem acerca da ética do cuidado, com total apagamento das desigualdades. E5: “A ética do cuidado tem características positivas que apenas seres humanos com o espírito de serviço podem garantir. Além disso, não há distinção de gênero, ideologia ou raça, o que torna o cuidado um chamado para servir”.^(19)^	E6: Discute a transversalidade da sexualidade nas maneiras de cuidar de enfermeiras. Reflete sobre as dificuldades das enfermeiras em lidar com situações que não se adéquam à heteronormatividade. E6: “No espaço público, a profissão foi, desde o início de sua institucionalização […], submetida ao forte esquema de neutralização dos corpos e interdição da sexualidade. A moral cristã, que abriu espaço inclusive para a crença na enfermeira como anjo assexuado, contribuiu enormemente para a negação do corpo erótico da enfermeira”^(20)^
E10: A pesquisa descreve a conciliação entre a vida profissional e familiar de mulheres enfermeiras que são mães, sem, contudo, criticar as injustiças de gênero advindas da sobrecarga do trabalho do cuidado. E10: “Mesclam-se neste fazer profissional muitos componentes da forma de se relacionar e do modo de ser feminino, o que faz com que a sensibilidade e o envolvimento pessoal com o sofrimento alheio acabem emergindo no âmbito de sua atuação profissional […]”.^(23)^	E7: A politicidade do cuidado subsidiou análises comparativas dos estereótipos das Evas-brasileiras e Marias-portuguesas com a enfermeira sexualizada ou santificada. As desigualdades de gênero das brasileiras em Portugal e das enfermeiras se inscrevem na moral judaico-cristã que reitera a subserviência do feminino ao masculino. E7: “Um desses estereótipos que cristalizam o imaginário da mulher […] é aquele que insiste em designá-la como uma cuidadora natural que, por sua natureza feminina, estaria destinada a se responsabilizar integralmente pelas atividades de cuidado.”^(1)^
E11: Concepção de cuidado como gesto de “amor” repleto de valores morais e religiosos presentes na historicidade da enfermagem, sem questionamentos. E11: “Tanto a empatia de Stein, como a simpatia de Scheler, estão intimamente ligadas ao nosso agir humano como ato de percepção das vivências dos outros, e […] levam o ser humano simpatizante ou empatizante a cuidar do outro com amor.”^(24)^	E8: A revisão sistemática identificou fragilidades na problematização das questões de gênero em 20 (57%) dos 35 (100%) artigos que se propuseram a analisar a relação entre cuidado, gênero e saúde. E8: “Esta situação exige problematizar a ordem social de gênero dentro de um contexto patriarcal em que o cuidado se contempla como uma função associada ao espaço doméstico e altamente feminilizado”.^(21)^
E12: As autoras identificam e ratificam nos discursos das docentes de enfermagem as ideologias conservadoras, os valores morais e os estereótipos de gênero que reforçam as injustiças sociais na profissão. E12: “A enfermagem também pode ser considerada uma qualidade que algumas pessoas possuem de forma inata que leva a uma predisposição especial, chamada vocação, para ajudar os outros […] é definida como uma atividade humana vinculada à mulher e relacionada com a maternidade, o cuidado dos filhos e a sobrevivência do ser humano. Esse instinto maternal é o que proporciona a motivação e o impulso necessário para cuidar”.^(25)^	E9: A revisão analisa 104 artigos da enfermagem a partir da epistemologia feminista. Preconceitos e cegueiras de gênero são evidenciados na produção científica da enfermagem oriunda de países da América do sul, América Central e Europa. E9: “Esse é um dos maiores problemas da pesquisa […]: considerar termos ou conceitos como constructos invariáveis e fixos. Especialmente na profissão, devemos rever a relação entre cuidado e gênero, a fim de ampliar os campos de leitura e abrir caminhos para entendimentos alternativos do que aparentemente não mudou por tantos anos”.^(22)^
E14: Sistematiza representações de cuidadores informais que associam o cuidado ao amor, à família, ao carinho, à proteção ou a missão adquirida, reproduzindo as ideologias e os estereótipos de gênero presentes na enfermagem. E14: “Cuidar de uma pessoa doente é uma situação que mulheres e homens vivenciaram em algum momento de suas vidas, que natural e espontaneamente assumem a responsabilidade, dedicam tempo e esforço a outros que não podem cuidar de si mesmos devido à idade, doença, invalidez ou deficiência para suprir suas necessidades”.^(27)^	E13: A pesquisa reflete sobre a relevância das mulheres cuidadoras para a sociedade e sua invisibilidade para as políticas públicas, a partir da categoria *care*, oriunda da epistemologia feminista. E13: “[…] É preciso mudar a representação segundo a qual as competências mobilizadas no trabalho do care são iguais ou um mero prolongamento das funções domésticas imputadas socialmente às mulheres”.^(26)^
E15: As experiências das cuidadoras mulheres são naturalizadas e essencializadas, sem reflexão crítica das questões de gênero. E15: “As mulheres aprenderam o papel de cuidadoras ao longo dos anos de socialização e aprimoraram suas habilidades participando do cuidado diário de seus filhos.”^(28)^	E21: Reflete sobre as implicações das questões de gênero na escolha dos cursos de enfermagem e pedagogia, repercutindo em desvalorização profissional. Ambiguidades entre a resignação e a resistência nos discursos das estudantes apontam perspectivas de enfrentamento das injustiças. E21: “Na Enfermagem […] persiste a feminização ‘tanto na qualificação universitária como nos níveis médio e técnico’ […] Assim, no caso das profissões feminizadas da saúde, mantém-se a relação entre ‘cuidado’ e ‘ação feminina’, num processo que naturaliza essas diferenças como atribuídas ao sexo feminino”.^(33)^
E16: O estudo aponta a divisão desigual e a precarização do trabalho do cuidado nas famílias pesquisadas, evidenciando as questões de gênero. Entretanto, não problematiza suficientemente as iniquidades, preferindo adjetivar como “supermulheres” as que assumem integralmente as múltiplas tarefas. E16: “O termo ‘supermulher’ […] define perfeitamente essas cuidadoras, que trabalham arduamente para manter múltiplos papéis, inclusive os de desenvolvimento pessoal, muitas vezes interrompidos devido a uma carga de trabalho excessiva […]”.^(29)^	E22: A pesquisa reflete sobre a sobrecarga das tarefas do cuidado para as mulheres, repercutindo em injustiças de gênero. Destaca a necessidade de democratizar a responsabilidade pelo cuidado no âmbito das políticas públicas de saúde e o papel da enfermagem nos processos de mudanças. E22: “O fato de o cuidado ter sido, e continuar considerado um tema da esfera feminina, reforça estereótipos de gênero sobre o papel de homens e mulheres na sociedade […]. Romper com os papéis socialmente designados para mulheres no cuidado pode ser a mudança que permite uma diferente distribuição do trabalho do cuidado.”^(35)^
E17: A pesquisa sistematiza determinismos biológicos, valores religiosos e estereótipos de gênero em quatro possíveis subtemas identificados para o cuidado: instinto de sobrevivência; olhar feminino; enfermagem; relação com um ser superior. E17: “[…] Para esse cenário específico, a mulher se destaca como receptora do ensino-aprendizagem do cuidado, destacando que essa função social lhe é praticamente exclusiva devido à sua capacidade de procriar”.^(30)^	E23: Questiona a reprodução dos estereótipos de gênero nos comportamentos maternais de docentes enfermeiras nas universidades, aprisionado numa velada obrigação moral do cuidado. Os reforços dos papéis de gênero são problematizados a partir da epistemologia feminista. E23: “A feminilidade hegemônica na enfermagem pode ser identificada como a imposição de normas comportamentais associadas ao feminino […] muitas vezes resultando em opressão horizontal. […] A imposição de comportamentos ostensivamente femininos pode despertar expectativas ‘maternais’ das mulheres no local de trabalho”.^(7)^
E18: Estudo quantitativo correlacional que reforça, sem questionamentos, a inferioridade das mulheres estudantes de enfermagem em relação aos homens, presumivelmente mais propensos ao pensamento crítico. E18: “Neste estudo, a feminilidade dos alunos foi positivamente associada ao comportamento de cuidar. Não houve correlação significativa, no entanto, entre feminilidade e pensamento crítico […] aqueles que relataram maior masculinidade apresentaram maior comportamento de cuidado e pensamento crítico do que seus colegas menos masculinos.”^(31)^	E25: O cuidado de enfermagem é concebido como uma prática política influenciada pela participação nos movimentos sociais de enfrentamento das desigualdades na profissão. E25: “No contexto dos serviços de saúde, são narradas como recorrentes algumas práticas opressoras, silenciadoras, que culpam, julgam e vitimizam, especialmente outras mulheres, e um cuidado familiar, romantizado […] Por essas características, o cuidado ofertado é entendido como inadequado e danoso”.^(37)^
E19: Foram extraídos dois fatores que sintetizam as concepções de cuidado das(os) graduandas(os), o primeiro, psicossocial, o segundo, técnico-profissional. O psicossocial é associado às mulheres, sem significância estatística, com ausência de questionamentos dos estereótipos de gêneros. E19: “Quanto à influência do gênero, nas mulheres pesquisadas, cinco das seis dimensões mais identificadas com o cuidado estavam relacionadas com o aspecto psicossocial, isso pode levar a pensar que as mulheres são mais preocupadas com aspectos relacionais e contextuais.”^(32)^	
E20: Estudo transversal descritivo que pressupôe o cuidado como atributo feminino. Portanto, os estudantes de enfermagem homens teriam dificuldades para a prática clínica. Essa associação entre cuidado feminino e imagem negativa da enfermagem é ratificada como pretensa autoevidencia essencializada. E20: “Vários pesquisadores descobriram que estudantes de enfermagem do sexo masculino encontram mais desafios no ambiente clínico do que estudantes do sexo feminino […], principalmente porque a enfermagem combina valores profissionais e femininos de cuidado”.^(34)^	
E24: A pesquisa reforça as violências de gênero no discurso científico da profissão, com estereótipos repletos de moralizações do cuidado, visto como gesto de altruísmo, amor e carinho. E24: “A forma de cuidar do outro é desvelada quando se preocupa em se colocar no lugar do outro, dando amor e carinho”.^(36)^	

Fonte: Elaboração própria.

Das 25 produções incluídas, a pesquisa qualitativa (n = 15; 60%) foi o método predominante, com presença de reflexões ou ensaios teóricos (n = 4; 16%), revisões narrativas ou sistemáticas de literatura (n = 3; 12%), bem como estudo quantitativo (n = 3; 12%). Quanto aos locais de publicação, concentraram-se em periódicos do Brasil (n = 18; 48%), da Inglaterra (n = 3; 12%), da Colômbia (n = 2; 8%), da Espanha (n = 2; 8%) e do México (n = 2; 8%). Os demais (n = 4; 16%) se distribuíram entre Cuba, Escócia, EUA e Canadá. Dentre as regiões globais, as produções se concentraram nas Américas (n = 19; 76%) e na Europa (n = 6; 24%). Acerca do ano de publicação, um maior número de artigos ocorreu no período de 2014 a 2019 (n = 14; 56%), seguidos dos intervalos de 1996 a 2013 (n = 6; 24%) e de 2020 a 2022 (n = 5; 20%). Na distribuição temporal dos artigos entre críticos (n = 11; 44%) ou reeditores do estereótipo (n = 14; 66%), não verificamos regularidades dignas de notas.

As pesquisas incluídas na revisão apresentam os seguintes objetos de estudo (Quadro 2): i-experiências, práticas, concepções, racionalidade e/ou aprendizados dos homens na enfermagem (E1; E2; E18; E20); ii-conceitos de cuidar ou de enfermagem para estudantes, enfermeiras(os) e/ou cuidadores de ambos os sexos (E12; E14; E17; E19; E24); iii-Tarefas e/ou éticas moralizantes do cuidado como feminino/maternal (E5; E10; E11; E15; E16); iv- Teorizações sobre a dimensão política, a perspectiva de gênero e/ou a sexualidade no cuidado na enfermagem (E3; E6; E7; E23; E25); v- Análises da perspectiva de gênero no ensino, na produção da enfermagem, no cuidado, na escolha da profissão e/ou nas desigualdades do trabalho do cuidado (E4; E8; E9; E13; E21; E22). Os sujeitos participantes das investigações incluíram as(os) estudantes de enfermagem (n = 968; 81,4%), distribuídos entre mulheres (n = 514; 53%), homens (n = 390; 40,2%) ou sem especificação (n = 43; 4,4%); estudantes mulheres dos cursos de pedagogia, enfermagem ou administração (n = 21; 2,2%); enfermeiras (n = 58; 79,4%); enfermeiros (n = 15;20,5%); cuidadoras mulheres (n = 66; 71,7%); cuidadores homens (n = 26; 28,2%); mulheres de baixa renda (n = 56;4,7%).

Embora a maioria dos artigos reproduza acriticamente o estereótipo da cuidadora natural (n = 14; 56%), as resistências epistêmicas (n = 11; 44%) criticam as desigualdades de gênero na profissão, constituindo-se em contraponto explícito. As evidências mapeadas foram agrupadas em seis categorias inter-relacionadas, sendo três para a reedição e três para a crítica do estereótipo da cuidadora natural. As categorias, com as respectivas referencias, são as seguintes: reedição: a) cuidado como essencialmente feminino (E1; E2; E10; E12; E15; E17; E18; E19; E20) ^(15,16,23,25,28,30-32,34)^; b) cuidado como chamado e serviço do amor (E5; E11; E24) ^(19,24,36)^; c) inocuidade/apagamento das desigualdades de gênero (E14; E16) ^(27,29)^; crítica: d) cuidado “inadequado e danoso” (E3; E4; E23; E25); ^(7,17,18,37)^ e) neutralização do gênero e dos corpos (E6; E8; E9)^(20–22)^; f) denúncia das opressões de gênero no trabalho do cuidado (E7; E13; E22) ^(1,26,35)^.

Dentre os resultados dos estudos que reeditam os estereótipos de gênero na enfermagem (n = 14; 66%), verificamos ratificações das concepções binárias de gênero (n = 5;20%), reprodutoras de estereótipos que rotulam a emoções como atributo feminino e a razão como masculina (E1; E2; E18; E19; E-20). Incluem-se nesse grupo os estudos quantitativos que analisam as experiencias dos homens na enfermagem (E1; E2; E20), com a hegemonia de pesquisadores masculinos na autoria. Outras investigações reproduziram conceitos de cuidado como sinônimo de amor, altruísmo, feminilidade ou como função procriadora, com evidente violência de gênero sobre as mulheres (E5; E11; E12; E14; E17). A essencialização da “cuidadora natural” como enfermeira, mãe e mulher se manifesta fortemente nas produções que se propuseram a refletir sobre o trabalho do cuidado (E10; E14; E15; E16). As produções que ratificam os estereótipos de gênero na enfermagem invisibilizam nas análises os recortes por população estudada (estudantes, profissionais de enfermagem ou cuidadoras(es), mulheres). Ou seja, eles tendem a tratar os sujeitos como um bloco homogêneo, sem maiores diferenciações de gênero, classe social, raça ou geração na discussão dos resultados.

Por sua vez, os artigos que criticam os estereótipos de gênero na enfermagem (n = 11; 44%), embora partam de objetos de estudo e sujeitos semelhantes, diferenciam-se pela problematização das análises realizadas. Alguns desses, sobretudo teóricos e qualitativos, assumem a centralidade do político no conceito e na prática da profissão (E3; E7; E25). Outros denunciam a naturalização do cuidado como essencialmente feminino, desvelando as injustiças discursivas (E4; E8; E9; E13; E21). A enfermagem como prática social, bem como os questionamentos dos sexismos e das desigualdades de gênero assumem destaque em parte das pesquisas críticas (E6; E21; E23). Nas pesquisas qualitativas dos artigos classificados como críticos, os recortes de gênero na população estudada são priorizados nas análises realizadas (E4; E6; E13; E21; e25).

## DISCUSSÃO

A concentração de artigos publicados em periódicos da região global das Américas (n = 19; 76%) pode ser explicada pelos mecanismos de busca da fase exploratória (BDENF via BVS) e pela preponderância de estudos da América do Sul na base SciELO. A despeito dessa limitação, a preferência por pesquisas clínicas e epidemiológicas dos periódicos da saúde, pouco afeitos às discussões epistêmicas do cuidado, pode ter contribuído para as escassas produções oriundas de outras regiões globais, merecendo aprofundamentos.

Face às características complexas do cuidado, as metodologias qualitativas presentes nos artigos são adequadas ao estudo do objeto, por permitirem o aprofundamento de singularidades. Entretanto, se considerarmos a interdisciplinaridade da epistemologia feminista à crítica dos estereótipos de gênero^(2–4,12)^, as poucas reflexões teóricas produzidas parecem comprometer o potencial crítico da ciência da enfermagem. Como sabemos, as reflexões teóricas dialogam melhor com as abordagens das ciências humanas e sociais, por serem oriundas desses campos. Nesse contexto, ressaltamos a capilaridade interdisciplinar nos estudos sobre gênero, haja vista a formação das pesquisadoras dos artigos críticos, quase todas com doutorado, pós-doutorado ou pesquisas na área de gênero, de ciências humanas ou sociais (E3; E4; E6; E7; E8; E9; E13; E21; E22; E23; E25),^(1,7,17,18,20,21,22,26,33,35,37)^ o que repercutiu nas produções das autoras. Assim, a coincidência entre os artigos críticos e a interdisciplinaridade na titulação das pesquisadoras indica a necessidade de ampliarmos a formação de enfermeiras para a análise crítica das questões de gênero na profissão, em diálogo com a epistemologia feminista.

O gênero como categoria de análise que problematiza o essencialismo da “Mulher” (letra maiúscula como denúncia ao totalitarismo e a rigidez semântica) foi a grande contribuição da epistemologia feminista às ciências^(2–4,12)^. Consequentemente, a perspectiva do gênero^(11)^ introduziu um relevante enfoque questionador aos resultados discursivos das investigações revisadas. Constatamos esse diferencial nas produções que assumiram a centralidade do político nas concepções e nas práticas da enfermagem (E3; E7; E25), nos que denunciaram a naturalização do cuidado como feminino (E4; E8; E9; E13; E21) ou naqueles que questionaram os sexismos e as iniquidades de gênero na profissão (E6; E21; E23).

Por seu turno, a hegemonia do positivismo, do tecnicismo e do produtivismo nas profissões da saúde^(38)^ – mantenedora do modelo biomédico, patriarcal, mercadológico e socialmente injusto para as mulheres^(2–5)^ – retroalimenta a insuficiência de teorizações críticas na área. Soma-se a isso o quase inexistente espaço dedicado às reflexões teóricas nos periódicos da saúde, pressionados pelo utilitarismo da ciência, o que desestimula pesquisas com pendor reflexivo e político sobre o cuidado. Esse cenário positivista, atrelado à historicidade da enfermagem imersa em ideologias sexistas, racistas e elitistas,^(39)^ conforma a insuficiência crítica dos artigos que reproduziram os estereótipos de gênero. Mais da metade dessas produções reafirma o cuidado como “essencialmente feminino”, sem qualquer filtro sobre o caráter opressivo para nós, mulheres.^(2,3,4,12)^

Perfazem os resultados das produções acríticas na enfermagem as concepções restritas ao binarismo de gênero (E1; E2; E18; E19; E-20), repletos de estereótipos moralizantes sobre o cuidado, repercutindo em violências para as mulheres, enfermeiras ou não (E5; E11; E12; E14; E17). As desigualdades do trabalho do cuidado^(2–4)^ são igualmente invisibilizadas no preconceito da “cuidadora natural”, por vezes associado a uma indistinção ideológica entre enfermeira, cuidadora e mãe (E10; E14; E15; E16). Como traço comum ao positivismo biomédico^(38)^, destacamos o total apagamento das diferenças de gênero, classe, raça ou geração no segmento populacional investigado, característica da pretensa neutralidade científica, geradora de iniquidades. Outrossim, observamos rígida demarcação nos papéis de gênero, com subordinação das mulheres, nos artigos que se propuseram a investigar a presença dos homens na enfermagem (E1; E2; E20), em sua maioria com autoria masculina. Este achado desvela a suposta isenção da ciência positiva e dos pesquisadores (notadamente homens), delineando as visões machistas e a autorreferencia dos discursos estereotipados da profissão. Dizendo mais claramente: pesquisadores homens tenderam a investigar a si próprio na enfermagem, com reprodução dos seus sexismos. Seria redundância dizer que as injustiças de classe, raça, gênero e geração conformam a historicidade da enfermagem, portanto os discursos estigmatizados apenas nos vituperam^(11,38,39)^.

Verificamos reforço do estereótipo de gênero nos discursos da enfermagem nas afirmações de que “aspectos do cuidado vieram naturalmente”^15^ para as “colegas mulheres” (E1)^(15)^, pois “uma vinculação de gênero entre cuidado e feminilidade” ^(16)^ é “cada vez mais […] necessária para transmitir o cuidado” (E2)^(16)^. Noutras vozes, lemos que “o modo de ser feminino”^(23)^ predispõe a uma “sensibilidade e envolvimento pessoal com o sofrimento alheio” (E10),^(23)^ posto que as mulheres “aprenderam o papel de cuidadora […] e aprimoraram suas habilidades participando do cuidado diário dos filhos” (E15).^(28)^ Nessas produções, a “Mulher” é vista como uma “receptora do ensino-aprendizagem do cuidado”,^(30)^ entendido como uma “função social” que seria “praticamente exclusiva devido a sua capacidade de procriar”^(30)^ (E17).

Nas categorias a) cuidado como essencialmente feminino e b) cuidado como chamado e serviço do amor, observamos o enraizamento de papéis de gênero que tipificam a mulher como sensível, emotiva e com pouco uso da razão, reeditado nos discursos das pesquisadoras. Essas enfermeiras defendem, sem filtros, que “as mulheres são mais preocupadas com aspectos relacionais” (E19).^(32)^ A mesma reprodução dos estereótipos de gênero se verifica numa pesquisa com estudantes de graduação em enfermagem, donde se conclui que “a feminilidade das alunas”^(31)^ está “associada ao comportamento do cuidar”^(31)^, enquanto que “maior masculinidade”^(31)^ se relaciona ao “pensamento crítico” (E18)^(31)^. Intrinsecamente imbricada nessas distorções, a visão do cuidado como um “chamado de serviço” do amor complementa o tom discriminatório dos discursos que nos agridem, sem qualquer filtro, manifestados pelas autoras. Por fim, na categoria c) inocuidade das desigualdades, as pesquisadoras apagaram as injustiças relacionadas ao gênero, visto como sinônimo de sexo, associado à exaltação estereotipada acrítica da “supermulher” (E16)^(29)^, como se fosse um fenômeno imutável e biologicamente determinado.

Destacamos um trecho que sintetiza o estereótipo da “cuidadora natural” sem parcimônia, reforçando violências simbólicas contra nós, mulheres enfermeiras, o qual merece profundas reflexões. Referimo-nos ao estudo que considera a enfermagem não como um trabalho, uma prática social ou uma profissão histórica, mas “uma qualidade que algumas pessoas possuem de forma inata”,^(25)^ a qual leva a uma “predisposição especial, chamada vocação, para ajudar os outros”^(25)^. A enfermagem, reiteram as colegas sem qualquer ressalva, seria “uma atividade vinculada a mulher e relacionada à maternidade”^(25)^, a qual “proporciona a motivação e o impulso necessário para cuidar” (E12).^(25)^

Conceber a enfermagem como um atributo feminino, numa visão essencialista imutável, assim como o cuidado como um “impulso” da maternidade, apenas acalenta práticas e discursos sexistas tidos como “naturais” – quando são socialmente produzidos para aprofundar as opressões de gênero na profissão. As autoras do estudo não deixam claro o que entendem por “impulso” (E12)^(25)^, quiçá por suporem “dada” e “autoevidente” a natureza essencialmente cuidadora da mulher, portanto isenta de quaisquer questionamentos. Tampouco discorrem sobre as repercussões nefastas para nós, enfermeiras, se considerarmos o cuidado, em vez de uma prática social situada em relações de poderes – portanto em situações estratégicas e flexíveis de domínios passíveis de mudanças pelas correlações de forças^(17)^ – uma espécie de pulsão freudiana, quer dizer, uma “representação psíquica e endossomática”^(40)^ incontrolável. Em suma, lemos nessa produção que a “qualidade inata” da enfermagem (e não o trabalho!) predisporia a ajudar os outros e estaria presente em nós, mulheres-objeto procriadoras, como uma força da natureza contra a qual não podemos lutar, contrapor, resistir ou negar criticamente, apenas aceitar passivamente.

Nada mais avesso às conquistas sociais e políticas que precisamos do que discursos germinadores e incubadores das desigualdades de gênero acerca da “cuidadora natural” nos nossos corpos^(11)^. Os equívocos, as iniquidades e os danos retroalimentados por esse tipo de ciência, surpreendentemente produzidos pela elite pensante da enfermagem, são prementes. Em flagrante contraste com as idealizações da profissão como “um chamado para servir” (E5)^(19)^, recordemos brevemente as péssimas condições laborais, as violências, as discriminações, as baixas remunerações, a ausência de piso salarial ou a fragilidade política da categoria nas lutas trabalhistas, entre outras injustiças contextualizadas na introdução^(1-7,9)^. Embora essas falas se expliquem estruturalmente na historicidade da profissão, permeada por ideologias conservadoras, sexistas e racistas que nos vituperam^(7,10,17,37)^, elas não determinam a totalidade dos discursos científicos da profissão. Outrossim, evidenciam uma maioria que não se percebe aprisionada no próprio discurso, tampouco identifica as repercussões hostis para nós, mulheres, na disseminação acrítica do estereótipo da “cuidadora natural” na enfermagem.

Como resistência no campo científico da profissão, outras produções problematizaram as desigualdades advindas dos estereótipos de gênero, agrupadas nas seguintes categorias: d) cuidado “inadequado e danoso”; e) neutralização do gênero e dos corpos; e f) denúncia das opressões de gênero no trabalho do cuidado. Representantes dessas classificações manifestaram um pensamento divergente amparado na epistemologia feminista, como dissemos acima. Tomamos de empréstimo a expressão do cuidado “inadequado e danoso” (E25)^(37)^, referida num dos artigos, para nomear a primeira categoria crítica. Nas argumentações, as investigadoras apontam o cuidado como uma prática social e política (E3)^(17)^; questionam as relações hierárquicas estabelecidas entre homens e mulheres no cuidar (E4);^(18)^ denunciam a “imposição de normas associadas ao feminino” (E23)^(7)^ e a naturalização da “relação entre cuidado e ação feminina”, assim como as “expectativas maternais das mulheres nos locais de trabalho”^(7)^. Os estudos críticos também desvelaram as narrativas opressoras que “culpam, julgam e vitimizam” (E25)^(37)^ as mulheres e o cuidado.

Por sua vez, nos artigos acerca da neutralização do gênero e dos corpos, segunda categoria crítica, as cientistas contestaram a interdição sexual e a negação nos corpos eróticos das enfermeiras (E6);^(20)^ problematizaram a “ordem social do gênero” (E8)^(21)^ do contexto patriarcal; assim como contestaram a concepção restrita de gênero nos estudos da enfermagem, a maioria centrada em “constructos invariáveis e fixos” (E9)^(22)^. Quer dizer, as pesquisadoras criticam a concepção de gênero que prevalece na ciência da enfermagem, a qual segue atrelada exclusivamente à concepção restrita de mulher ou apaga as diferenças hierárquicas (E9)^(22)^. Na terceira categoria crítica, os artigos denunciam as opressões de gênero no trabalho do cuidado, falsamente justificados no estereótipo da “cuidadora natural” (A7)^(1)^. As estudiosas do *care* na enfermagem defendem mudanças nas representações do trabalho das mulheres como “prolongamento das funções domésticas” (E14)^(27)^, uma vez que a “ruptura com os papéis socialmente designados para as mulheres” (E22)^(35)^ contribui para uma distribuição mais justa do trabalho do cuidado.

De fato, uma das relevantes discussões da epistemologia feminista argumenta que a relação entre cuidado e capitalismo passa pela dimensão reprodutiva do trabalho, cuja responsabilidade recai desigualmente para as mulheres (ou “servas do capital”)^(2)^. Ou seja, num contexto de economia global, o trabalho produtivo, gerador de acumulação mediante valores de troca, precisa indissociavelmente da dimensão reprodutiva, daquela que produz a força de trabalho saudável para ser explorada, forjada nas atividades para manutenção da vida (tarefas domésticas, cuidados de saúde, das crianças, dos idosos, de bem-estar, outros). Noutras palavras, na complexidade das relações produtivas do cuidado, o capitalismo não sobrevive sem o trabalho desigualmente atribuído às mulheres. Nessa seara, a subversão da lógica patriarcal de distribuição das tarefas do cuidado pode tornar mais equânime a relação entre os gêneros, repercutindo na democracia^(2-4)^.

Numa similitude para a saúde, haja vista a maioria quantitativa das trabalhadoras da enfermagem, a oferta dos serviços depende visceralmente das enfermeiras e das técnicas. A despeito dessa sustentação majoritária, o viés discriminatório que voluntariamente proclamamos dociliza nossos corpos, entorpece nossas reflexões, silencia nossas vozes e mina nossa força política para modificar as indignas condições de trabalho. Não obstante, as contribuições deste e de outros estudos na identificação das resistências críticas dentro do campo epistêmico da enfermagem podem desequilibrar esse pêndulo em nosso favor.

Nesse contexto, as estratégias para o enfrentamento dos estereótipos de gênero na enfermagem passam por uma maior articulação entre a pesquisa, a educação, a organização política e a prática. No âmbito da formação de enfermeiras e equipe, urge incluirmos a perspectiva de gênero na problematização crítica dos discursos endógenos que nos aprisionam em opressões simbólicas, posto que eles apenas alargam as violências, a desvalorização salarial e as condições indignas de trabalho. A epistemologia feminista constitui um enfoque necessário aos conteúdos disciplinares e extracurriculares, especialmente naquelas que dialogam com a historicidade, o cuidado e o exercício profissional. Mais ainda, a pesquisa e a ciência da enfermagem, articulada ao ensino e a extensão, pode dialogar melhor com os referenciais feministas na produção dos pensamentos críticos e dos engajamentos políticos na enfermagem. No âmbito das associações de classe, precisamos problematizar de maneira aprofundada, nos vários fóruns de discussão, o quão injusto, cruel e opressivo são os discursos ideológicos que reproduzimos, ampliando nosso poder de enfrentamento das iniquidades. Num curto prazo, os resultados desse artigo podem subsidiar discussões problematizadoras sobre o cuidado e os estereótipos de gênero na produção científica da enfermagem, em cenários de aprendizagem diversos, entre estudantes de enfermagem, docentes, enfermeiras e enfermeiros.

### Limitações do Estudo

Por não termos contactado as autoras dos artigos não disponibilizados na internet, alguma referência importante pode ter sido suprimida do estudo. A exclusão da literatura cinzenta pode ter diminuído a quantidade das produções críticas dos estereótipos de gênero na enfermagem, presentes em dissertações de mestrado e teses de doutorado.

## CONCLUSÃO

A produção científica da enfermagem sobre o cuidado, em sua maioria, reproduz estereótipos de gênero e as idealizações que reforçam as opressões sobre as mulheres da profissão. Nesses estudos, preponderam discursos que cristalizam a “cuidadora natural” e assinalam um cuidado vinculado ao feminino, como se fosse “um chamado e serviço do amor” – nunca uma relação social de poderes em disputa, disruptiva.

Em contraponto, as resistências críticas de enfermeiras cientistas denunciam a naturalização do cuidado feminino como “inadequado e danoso”, por perpetuar as opressões de gênero. Essas produções se amparam na epistemologia feminista e utilizam o gênero como uma dimensão analítica questionadora dos binarismos, das interdições sobre o corpo, das falsas neutralidades e dos apagamentos dos sexismos. Em consonância com esses estudos, perguntamos o quanto essas questões integram o nosso fazer científico para repercutir na formação crítica de enfermeiras e de técnicas. Ou o que aconteceria se acumulássemos práticas discursivas para confrontar os poderes mercadológicos, misóginos, biomédicos e patriarcais por detrás de cada cuidado “bondoso”, ou seja, daquele do falso “impulso” (feminino) para subservir.

Haja vista a centralidade do cuidado para a enfermagem, os achados desta revisão sinalizam a necessidade de ampliar a autocrítica sobre os discursos científicos da profissão, de forma a desvelar padrões sexistas que nos violentam de forma endógena, invisível e acrítica. As implicações deste estudo para a pesquisa em enfermagem apontam para uma lacuna nas produções científicas sobre o cuidado a partir da perspectiva de gênero, com incipiente poder de crítica a partir da epistemologia feminista. No âmbito das práticas da enfermagem, a reedição de estereótipos de gênero no discurso das(os) pesquisadoras(es) enfermeiras(os), além de manter condições indignas de trabalho, obstaculiza a conquista de direitos, a autonomia e a valorização profissional.
